# A mechanistic model for spread of livestock-associated methicillin-resistant *Staphylococcus aureus* (LA-MRSA) within a pig herd

**DOI:** 10.1371/journal.pone.0188429

**Published:** 2017-11-28

**Authors:** Anna Irene Vedel Sørensen, Nils Toft, Anette Boklund, Carmen Espinosa-Gongora, Kaare Græsbøll, Jesper Larsen, Tariq Halasa

**Affiliations:** 1 National Veterinary Institute, Technical University of Denmark, Lyngby, Denmark; 2 Microbiology and Infection Control, Statens Serum Institute, Copenhagen, Denmark; Wageningen Universiteit, NETHERLANDS

## Abstract

Before an efficient control strategy for livestock-associated methicillin resistant *Staphylococcus aureus* (LA-MRSA) in pigs can be decided upon, it is necessary to obtain a better understanding of how LA-MRSA spreads and persists within a pig herd, once it is introduced. We here present a mechanistic stochastic discrete-event simulation model for spread of LA-MRSA within a farrow-to-finish sow herd to aid in this. The model was individual-based and included three different disease compartments: susceptible, intermittent or persistent shedder of MRSA. The model was used for studying transmission dynamics and within-farm prevalence after different introductions of LA-MRSA into a farm. The spread of LA-MRSA throughout the farm mainly followed the movement of pigs. After spread of LA-MRSA had reached equilibrium, the prevalence of LA-MRSA shedders was predicted to be highest in the farrowing unit, independent of how LA-MRSA was introduced. LA-MRSA took longer to spread to the whole herd if introduced in the finisher stable, rather than by gilts in the mating stable. The more LA-MRSA positive animals introduced, the shorter time before the prevalence in the herd stabilised. Introduction of a low number of intermittently shedding pigs was predicted to frequently result in LA-MRSA fading out. The model is a potential decision support tool for assessments of short and long term consequences of proposed intervention strategies or surveillance options for LA-MRSA within pig herds.

## Introduction

Methicillin-resistant *Staphylococcus aureus* (MRSA) are a group of *S*. *aureus* that have acquired the *MecA* or *MecC* gene, which make them resistant to most β-lactam antibiotics [1]. Three main groups of MRSA exist. Hospital-acquired MRSA (HA-MRSA) was identified in the late 1980s and was the dominant source of MRSA infections until community-acquired MRSA (CA-MRSA) emerged in the mid-1990s [2]. Livestock-associated MRSA (LA-MRSA) in humans was identified for the first time in the Netherlands in 2005 [3,4].

The pig population is the main reservoir for LA-MRSA, but LA-MRSA are also found in a wide range of other animals, including cattle, horses, chickens, turkeys, rats, dogs and cats [2,5–9]. The majority of LA-MRSA strains harbor *tetM* and sometimes also *tetK* [10,11], which causes resistance to tetracyclines, the most used antimicrobial group in the Danish pig production [5]. Other resistance genes are often present in LA-MRSA as well, in addition to the zinc resistance determinant *czrC* [12–14].

Like other *S*. *aureus*, LA-MRSA is an opportunistic pathogen in humans, where it colonizes the anterior nares. Only a minority of humans exposed to LA-MRSA become carriers and of these most will be asymptomatic carriers. However in those susceptible, LA-MRSA is capable of causing a large variety of conditions, ranging from mild skin and soft tissue infections to more severe conditions, e.g. pneumonia, meningitis and septicemia [15].

The majority of humans identified as LA-MRSA carriers have either been farm workers, veterinarians or members of households including farm workers/veterinarians. Thus, the main routes of transmission are assumed to be direct animal contact or direct exposure to air within the barns or indirect animal contact through close contact with individuals having direct animal contact [4,16].

In recent years, LA-MRSA has received considerable attention in Denmark due to an increased number of individuals being identified as carriers of this pathogen, albeit this partly could be explained by a revision of the national sampling guidelines causing more people at high risk of being carriers to be tested. In 2015, LA-MRSA CC398 accounted for 18% (208/1,147) of all reported MRSA infections in Denmark [5]. Nevertheless, compared to other European countries, the overall MRSA prevalence in Denmark remains low [17]. However, with 30.9 million pigs slaughtered or exported in 2015 [5], the national pig population constitute a potential LA-MRSA reservoir of a considerable size. In the last screening conducted by the Danish Veterinary and Food Administration in 2016, LA-MRSA was detected in 88% of randomly selected production herds [18].

Before the implementation of a national control strategy can be decided upon, it is essential to understand how LA-MRSA spreads and persists within a pig herd, once it is introduced. For that purpose, we built a mechanistic Monte Carlo simulation model for spread of LA-MRSA within an integrated pig herd. This model can be used for studying the colonization dynamics of LA-MRSA and for assessing the short and long term consequences of proposed interventions against LA-MRSA at farm level in terms of efficiency and cost-effectiveness, and thus be used as decision support before the implementation of these. It can also be used for investigating how a cost-effective surveillance system for early detection of LA-MRSA on a farm and subsequent decontamination could be designed. To the best of our knowledge, this is the first individual-based simulation model for spread of LA-MRSA within a pig herd to be described.

The objective of this study is to develop a model to aid a better understanding of the dynamics of LA-MRSA spread within an integrated pig farm following different routes of introduction.

## Materials and methods

A dynamic mechanistic Monte Carlo simulation model for the spread and persistence of LA-MRSA within a pig herd was built in R version 3.2.2– “Fire Safety” [19]. The model is individual-based and uses discrete time-steps set to one day each.

### Herd model

#### Herd type and size

The herd model represents an integrated sow herd with all age groups from farrow-to-finish at one site. We aimed at modelling a typical Danish medium-sized production farm, comprising 500 sows and with an annual production of 15,400 slaughter pigs [20,21]. Since the majority of Danish integrated herds purchase gilts from other herds [22], we included purchase of these in the model. It was assumed that the herd relied solely on artificial insemination and thus there was no influx of boars.

#### Farm design

**Fig 1:**
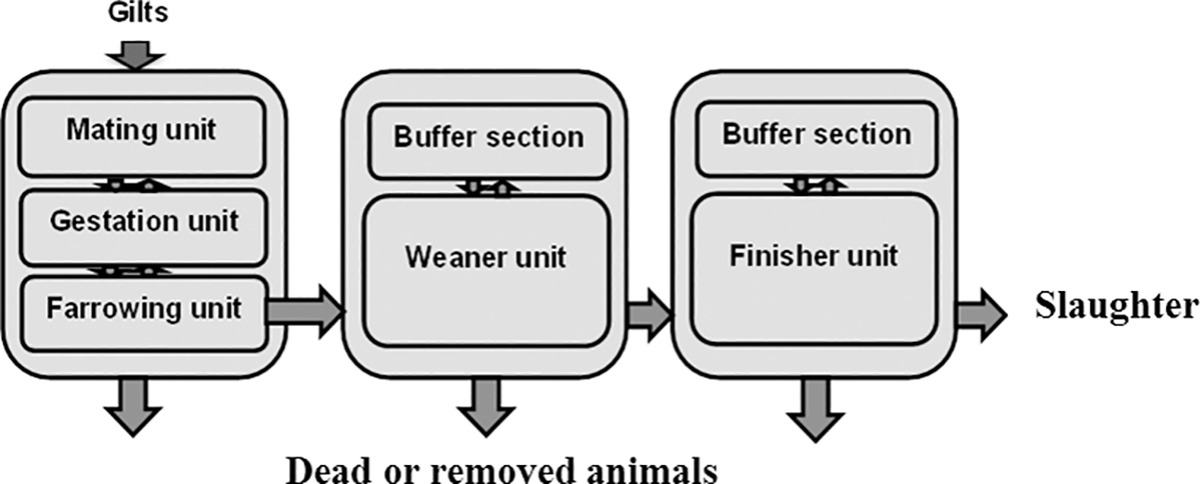
Flow between stable units in a simulated Danish integrated herd.

In the model, pigs were housed in five different units; a sow barn containing three units: 1) a mating and control unit, 2) a gestation unit, 3) a farrowing unit; and two separate barns containing 4) a weaner unit and 5) a finisher unit, respectively (Fig 1). The weaner unit and the finisher unit both included a buffer section, where pigs were housed if they were not ready to be moved to the finisher unit or to be sent for slaughter together with the rest of their batch. With the exception of the gestation unit, each stable unit was divided into several different sections (rooms), where each section housed a varying number of pens depending on the age group (Table 1). Pigs were moved between the units according to age (Table 1). Gilts awaiting first insemination were housed in a separate section in the mating unit, whereas sows awaiting return to oestrus before re-insemination were housed together with other sows in the mating unit awaiting service. In the farrowing unit, it was assumed that sows selected as nursery sows (foster dams) were moved to the section where the piglets to be nursed were born.

**Table 1:** Housing in different stable units in a hypothetical farrow-to-finish pig herd with 500 sows.

	Mating unit	Gestation unit	Farrowing unit	Weaner unit	Finisher unit
Time spent in the unit	Day 1–33 in each sow cycle	Day 34–113 in each sow cycle	Sows: Day 114–147	Day 29–77	Day 78-slaughterage
			Piglets: Day 1–28		
Pigs in the unit	Sows, gilts	Gestating sows	Sows + piglets	Weaners	Finishers
Sectioning in the unit	Full	None	Full	Full	Full
System within the unit	Individual housing of sows	Loose-housing	Individual housing with piglets	Max. 30 pigs per pen	Max. 15 pigs per pen
	Max. 5 gilts per pen	One pen per batch			
No. of sections	5 + 1 for gilts	1	5	8 + 1 buffer	14 + 1 buffer
No. of pens per section	40 (12 for gilts)	12	35	14 (3 in buffer)	24 (10 in buffer)
Snout contact btw. neighboring pens	Yes	Not relevant	Yes	Yes	Yes

#### Production cycle

We simulated a farm with weekly batch production in 21 sow batches and all-in/all-out production on section level. One full sow production cycle (mating, gestation, farrowing and nursing) was assumed to take 147 days (S1 Fig). At start of simulation each sow batch consisted of 23–26 sows of different ages and parities, which were at the same stage in the sow cycle (S1 Table).

#### Re-insemination of sows

It was assumed that sows were ready to be inseminated five days after weaning. The probability of insemination failure was 0.12, where sows to be re-inseminated were selected through a binomial process [23]. For simplification, it was assumed that both return of oestrus and lack of pregnancy without return to oestrus would be discovered three weeks after insemination. Consequently, re-insemination of the majority of the open sows would be attempted, while the remaining ones would be selected for strategic culling based on parity. The probabilities of re-insemination being attempted are given in S1 Table. Re-inseminated sows would be permanently moved to the sow batch, where the other sows in the batch had been inseminated in the same week as them.

#### Use of nursery sows (foster dams)

Litter size (live-born piglets only) was drawn from a normal distribution and rounded into integers (S2 Table). For litters consisting of more than 14 piglets, the surplus piglets were foster bred by a nursery sow. A two-step nursery sow system was used; surplus piglets from several sows were given to a sow that until then had been nursing her own 8-day old piglets. The 8-day old piglets from this sow were moved to a second sow, whose own piglets were ready to be weaned. After nursery piglets had been weaned, nursery sows would remain part of the sow batch to which they were moved upon selection as nursery sows.

#### Removal of sows

Four different processes for culling/deaths of sows were incorporated in the model. Strategic culling took place either immediately after weaning or after insemination had failed to result in pregnancy. Deaths or emergency culling could occur anytime with a probability depending on parity and current stage in the sow cycle (S3 Table). In all processes, the probability of a sow getting removed increased with the number of parities. At the very latest the sows were culled after their eighth litter had been weaned.

#### Replacement of sows

Gilts were included in the herd at least seven weeks prior to their first insemination, which was assumed to take place, when they were at least 243 days old, which is within the age range generally recommended in Denmark (230–260 days) [24]. The size of the gilt stock on the farm was evaluated on a weekly basis, and new animals were added when needed. Three days prior to insemination, the size of the sow batch ready for insemination was evaluated, and if it consisted of less than 23 pigs, new gilts were added to reach this number, in order to maintain a constant supply of piglets.

#### Weaning and placement into pens

Piglets were weaned after four weeks. Since it is common practise on many Danish farms to sort the pigs according to size, piglets from different litters were randomly mixed during movement to the weaner unit, and again upon entering the finisher unit. It was assumed that pigs were selected for slaughter twice a week, and that 20% of the pigs in the batch would be ready for slaughter earlier or later than the rest of the batch (S4 Table). In the event of a stable section running full, for simplicity and in order to ensure stability of the model, it was assumed that the surplus pigs was sold or slaughtered.

#### Use of buffer sections in the weaner and finisher unit

It was assumed that for a certain proportion of the weaners and finishers, the pigs would not be big enough to follow the rest of the batch when they were moved from the weaner to the finisher unit or sent for slaughter. These pigs were assumed to be moved to a buffer section within either the weaner or finisher unit, where they might be mixed with pigs from other batches. In the weaning unit, 20% of weaners scheduled to leave the unit would remain in the buffer unit for another week. These pigs were sampled randomly and each pig could be repeatedly selected for an additional one week stay in the buffer stable again in up to three consecutive samplings. In the finisher unit, the remaining pigs in a section would be moved to the buffer stable, if the number of animals left within a section decreased to below a certain threshold (calibrated to 150 animals) and the animals were at least 158 days old.

#### Removal of piglets, weaners and finishers

The probability of death or removal of piglets was age-dependent with a higher probability of removal during the first days of their lives (S5 Table). For weaners and finishers, we assumed a constant daily probability of death or removal (S5 Table).

### Epidemic model

#### Definitions

In the present model, we did not take into consideration, whether the pigs are truly colonized by LA-MRSA or only contaminated. Instead we used the terms intermittent shedder (IS) and persistent shedder (PS) to define a pig, which either temporarily or permanently harbours LA-MRSA in the nasal cavity in levels detectable by the method used by Broens et al. 2012 [25], and is able to spread LA-MRSA to another pig. For simplification, it was assumed that all pigs harbouring LA-MRSA in the nasal cavity were equally likely to spread it to other pigs. It was also assumed that ‘recovery’ implies that the animal is no longer shedding LA-MRSA, but that no immunity towards re-acquisition was acquired. All parameters have been based on data for LA-MRSA CC398, when available in the literature. Where no published data for CC398 were available, parameters have been based on LA-MRSA belonging to other clonal complexes or general estimates for *S*. *aureus*. In the rest of this text, MRSA will refer to LA-MRSA unless stated otherwise.

#### Structure

**Fig 2:**
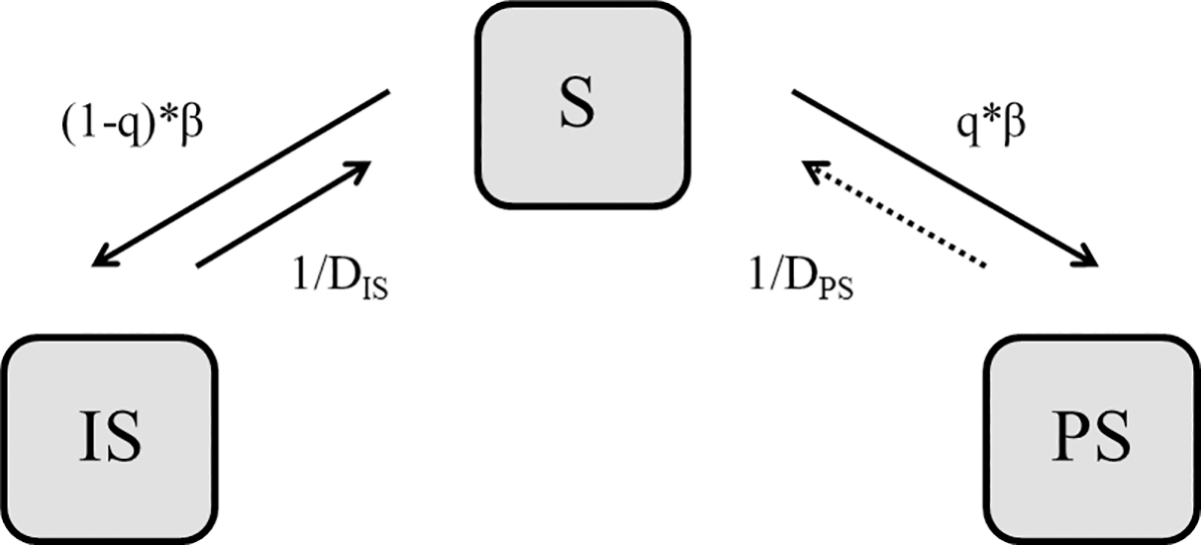
Infection model for MRSA. S = Susceptible, IS = Intermittent shedder, PS = Persistent shedder, β = Overall transmission rate, q = fraction of shedders becoming persistent shedders, D_IS_ = Duration of shedding for intermittent shedders, D_PS_ = Duration of shedding for persistent shedders, D_PS_ >> D_IS_.

The infection model was structured as an SIS compartmental model with one susceptible stage and two separate infectious stages for IS and PS (Fig 2) and one overall transmission rate (β) for going to one of the infectious stages. The probability of pigs becoming PS was assumed to depend on the infectious pressure in their environment as well as host-related factors. A proportion (mean: 24%) of randomly selected pigs (equal to maximum q on Fig 2) was assigned the potential to become PS (assumption based on [26]). The probability of these pigs actually becoming PS after exposure (q) was dependent on the prevalence of pigs shedding MRSA in the section, where they were housed. For simplicity, we introduced a prevalence threshold (most likely value: 70%), where the threshold level was estimated from [26]. Two different probabilities were applied for below (most likely value: 10%) or above the threshold (most likely value: 75%) (S6 Table). Both probabilities and the prevalence threshold were drawn from pert distributions. The proportion of pigs with the potential to become persistent shedders was sampled from a normal distribution (S6 Table). It was assumed that pigs stopped shedding after a given number of days (D_IS_ or D_PS_) and went back to being susceptible. However for the vast majority of the PS, this does not happen.

#### Transmission parameters

The transmission rates for MRSA used in the model were based on the results of a transmission study conducted on four Dutch farms, where pigs were followed from farrow to finish [25] (S7 Table). In this study, the transmission rates were determined separately for pre-weaning and post-weaning pigs. In our model, the transmission rates estimated in the Dutch study [25] for post-weaning pigs were used both for weaners and finishers, as well as for transmission between gilts and sows in the mating unit or gestation unit, whereas the pre-weaning rates were used for transmission between pre-weaning pigs and for transmission from sow to offspring after day 1.

Due to the uncertainty related to the transmission parameters, we decided to simulate all scenarios three times using one of three different sets of transmission rates each time (S7 Table) in an attempt to model both worst and best case scenarios for every situation plus a scenario in between. Since the transmission rates among pre- and post-weaning pigs were also determined both with and without use of risk antimicrobials in the Dutch study [25], the highest and the lowest set of rates used in our model were based on their results. Use of risk antimicrobials was defined on pen level as at least one pig within the pen receiving tetracyclines or β-lactam antibiotics within a time interval between samplings [25]. The set of medium rates were created based on the average values of the two other sets, to represent a farm with a moderate use of antibiotics, relative to the two other levels (S7 Table). The transmission rates used for the individual iterations were sampled from pert distributions.

Transmission of MRSA from sows to new-born piglets on the day of farrowing was modelled as a simple probability of the offspring being MRSA positive given it had been born by an MRSA positive dam, where the probability was sampled from a pert distribution with a probability interval based on the results of a study of the effect of sow status on piglet colonisation age [27] (S7 Table). The probability of piglets born by an MRSA-negative sow becoming MRSA shedders during their first day of life changed depending on the presence or absence of MRSA shedders within the section. If no shedders were present, the probability was set to zero. Otherwise, a probability drawn from a pert distribution (based on [27]) was used (S7 Table). After the first day in the piglets’ life, the pre-weaning transmission rates estimated by [25] were used both for spread of MRSA between piglets being nursed by the same sow and for spread between the sow and its piglets.

Four different transmission routes for spread of MRSA between pigs were modelled: 1) Transmission within the same pen; 2) Transmission between pens within the same section; 3) Transmission between sections; 4) Transmission between stables. The transmission rates for within pen and between pen transmission were based on data from [25] (S7 Table; calculations described in S1 Appendix). No data for transmission between sections or stables were available, and these rates will naturally depend on local conditions, e.g. the design of the stables, ventilation system and biosecurity measures in place. In our model, the spread between sections and stable units were assumed to be a fraction of the between-pen transmission rate used in the scenario in question. It was assumed that more handling of animals would take place in the farrowing and mating units compared to in the other units. Therefore, the fraction of spread between pens applied for spread between sections within these units was assumed to be 0.20, while 0.15 in the other units (S7 Table). We did not differentiate between spread from different sources e.g. pigs, humans, equipment, dust.

Transmission between pigs within a given unit was assumed to be density-dependent, i.e. the contact rate between pigs is assumed to be dependent on the number of pigs within the entity (pen, section or unit). The probability of a given pig (pig_j_) becoming an MRSA shedder as a result of contamination from pigs within the entity, where it was housed, was given by:

$${\mathrm{Prob}}_{E\left(j\right)}=1-e^{-\beta_{Ej}*\Delta T*\frac{I_{Ej}}{N_{Ej}}},$$ (1)

where β_Ej_ is the within entity transmission rate for transmission of MRSA during a time step; I_Ej_ is the number of infectious pigs within the entity, where pig_j_ is housed, during that time step; ΔT is the difference in days between the current and previous time step (which was always equal to 1); and N_Ej_ is the total number of pigs within the entity, where pig_j_ is housed during that time step. Entity can be equal to: pen (WP = spread within pen); section (BP = spread between pens within the same section); unit (BSe = spread between sections within the same unit) or farm (BSt = spread between stable units within the same farm).

For each susceptible pig (pig_j_) the total daily probability of becoming an MRSA shedder (Tot_ProbInf(j)_) during a time step was calculated as:

$$Tot_{ProbInf\left(j\right)}=1-\left(\left(1-Prob_{WP\left(j\right)}\right)*\left(1-Prob_{BP\left(j\right)}\right)*\left(1-Prob_{BSe\left(j\right)}\right)*\left(1-Prob_{Bst\left(j\right)}\right)\right),$$ (2)

where Prob_WP_, Prob_BP_, Prob_BSe_, and Prob_BSt_ are the probabilities of becoming shedder as a result of within-pen, between-pen, between-section and between-stable spread, respectively.

Based on human studies we assumed that IS and PS also constituted two distinct groups in pigs with distinctly different durations of shedding [28]. Duration of shedding for IS was sampled from a pert distribution based on a transmission study carried out under experimental conditions [29] (S2 Table). It was assumed that PS had no probability of recovery during the first 100 days of shedding and thereafter a 0.01 probability of recovery (selected for simplification, based on periods of 84 and 154 days used for carriage classification in two human studies [28,30]). The assumption of no recovery was based on the relatively short lifespan of slaughter pigs and reports of humans carrying the same *S*. *aureus* strain for up to eight years [31].

### Model output and validation

#### Model run

The model was run for six years following a burn-in period of four years before MRSA was introduced. The length of the burn-in period was based on the time needed for the number of pigs to stabilise, after simulation had been initiated.

The minimum number of iterations needed was determined, based on when convergence had been reached, assessed as the number of iterations needed for the variance of the total prevalence of MRSA-positive pigs in the herd at the end of run-time to stabilize (S2 Fig). Based on this 200 iterations were assessed to be enough to reach convergence. Nevertheless, the model was run in 500 iterations per scenario, in order to ensure higher stability of the outcomes, because the model was run with different sets of transmission rates and we expected the stability to vary.

#### Introduction of MRSA

In order to investigate spread and persistence of MRSA following different scenarios of MRSA introduction in an MRSA-free herd, various introductions were simulated: 1) Single or multiple introductions (fortnightly repeated for three months); 2) Introductions in different age groups (gilts, weaners or finishers); 3) Introductions of various numbers of shedders (1, 3, 10, 50 or 100) and; 4) Introduction of IS or PS. Not all combinations of these four parameters were modelled and only the most interesting results are presented in this paper.

#### Output parameters

The following model output parameters were used for comparison and visualisation of the scenarios modelled: 1) Development of the prevalence of MRSA shedders over time; 2) Proportion of iterations where MRSA fades out following introduction and time before fade-out; 3) MRSA prevalence in the different stable units.

#### Validation

Before the epidemic model was added, the herd model was validated using the rationalism method (assessing whether the output changed as expected following changes in the input values) and the tracing method (following individual animals over time) [32]. Production outputs simulated in the herd model were compared to production data from a sample of Danish herds [23]. The majority of the code for the model was also verified by an expert/another programmer (face validity).

#### Sensitivity and robustness analysis

The sensitivity analysis mainly focused on assessing the effect of duration of shedding, and how the status as IS or PS was assigned.

The pert distribution used for duration of shedding for IS was altered from a most likely value of 7.5 days (min = 1 day, max = 26 days) to 18 days (min = 6 days, max = 29 days) based on data from the same study as the original value [29], where a different definition of when pigs were to be considered MRSA positive was applied (S2 Table). The ranges of values obtained based on either definition were both consistent with data from another study, where the duration of carriage ranged from 1–39 days [33].

Spread following introduction of one IS gilt were modelled with two different modifications of the concept of how to select pigs to become IS or PS: 1) All pigs will become IS upon exposure (no PS); 2) Whether pigs become IS or PS is solely determined by host-factors (no influence of the prevalence of MRSA shedders in the room).

Since all scenarios had already been modelled using three different sets of transmission rates, only one additional set of transmission rates was introduced during sensitivity analysis. In this set, the same transmission rates were used for both pre- and post-weaning pigs. The transmission rate used for within-pen transmission was sampled from a pert distribution based on values calculated from the results of an inoculation study [33], where mean values (for three groups of pigs) of the reported transmission rates and the lower and higher 95% confidence interval limits were used as the most likely value, minimum and maximum, respectively (S2 Table). Since only within-pen transmission rates were reported, it was assumed that the ratio of between-pen and within-pen transmission was the same as for the transmission rates used in the standard scenario. As a result, the between-pen transmission rate was calculated by multiplying the within-pen rate with the average ratio of between-pen and within-pen transmission rates in the lowest and highest sets of transmission rates (S2 Table).

The robustness of the model was assessed by changing more than one parameter at a time (S8 Table).

## Results

### Validation

Any unexpected output discovered using the tracing and rationalism method or during expert validation were further investigated and followed by corrections of the code, when needed.

Various production parameters were included in the herd model output and compared to real-life production data from swine Danish herds, in order to externally validate the model and check if the parameters were appropriately calibrated (S9 Table). The model output and real-life data generally had good agreement.

### Spread of MRSA

**Fig 3:**
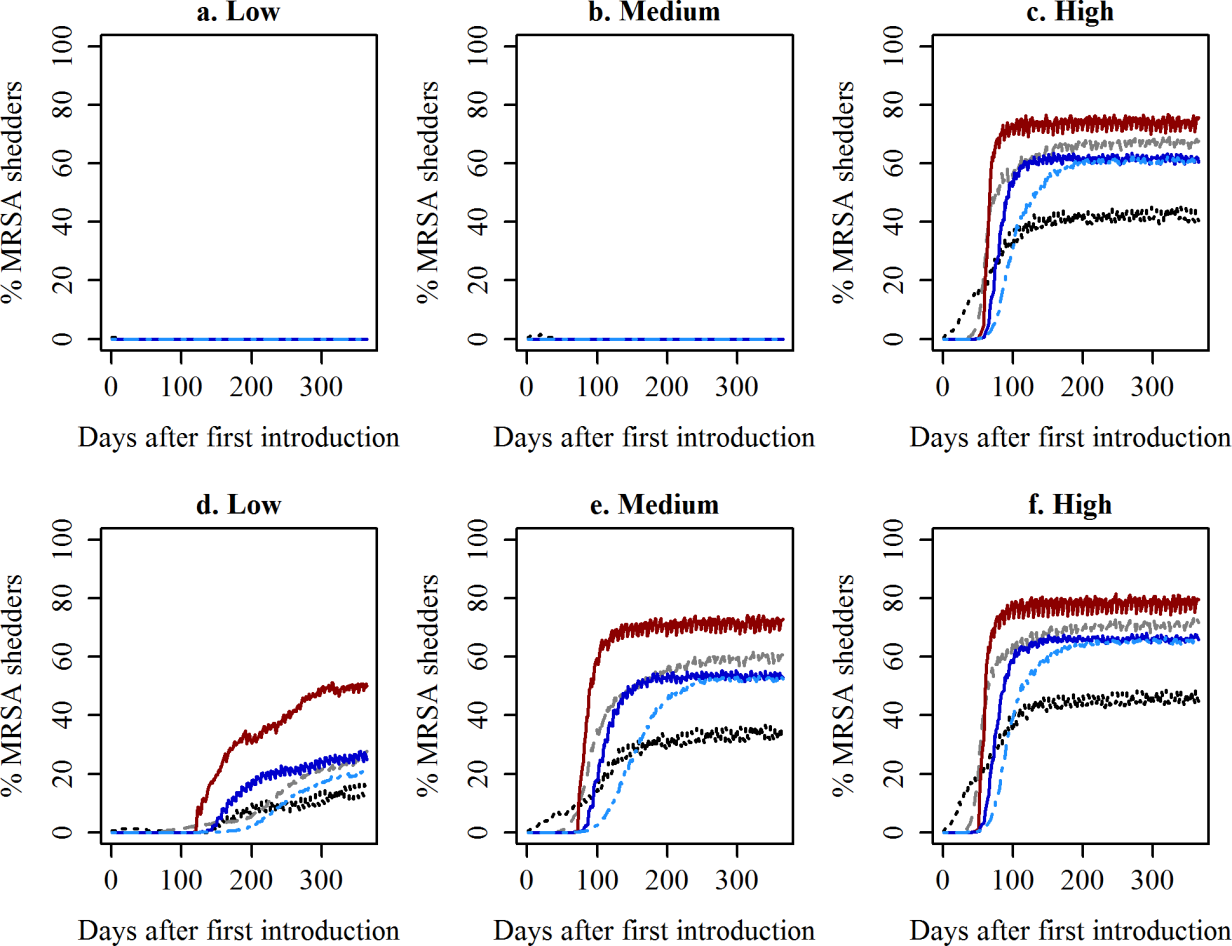
Development in the median prevalence of MRSA shedders following introduction of one MRSA shedding gilt. Predicted median prevalence over time following introduction of one intermittently (a-c) or persistently shedding gilt (d-f), when using low (a+d), medium (b+e) or high (c+f) transmission rates. Mat = Mating unit, Gest = Gestation unit, Farr = Farrowing unit, Wean = Weaner unit, Fini = Finisher unit.

When low or medium transmission was assumed, introduction of MRSA by one IS gilt was in most cases predicted to result in MRSA fading out (Fig 3A). When high transmission were assumed, then based on the median values, spread from the mating unit to other units was not observed, before enough time had elapsed for some of the gilts to be pregnant and be moved to the gestation unit (Fig 3B and 3C). After introduction in the farrowing stable the number of shedders saw a marked increase, followed by spread into the weaner unit and later into the farrowing unit (Fig 3). MRSA mainly seemed to be following the routes of the animals. However, if MRSA was introduced in the weaner unit or finisher unit, the simulations indicated that spread to the sow units was still likely to occur, despite animals not being moved backwards (S3 and S4 Figs). The later in the production process MRSA was introduced (gilts → weaners → finishers), the slower spread and thereby longer time before the prevalence in the stables units stabilized (Fig 3 and S3 and S4 Figs).

**Fig 4:**
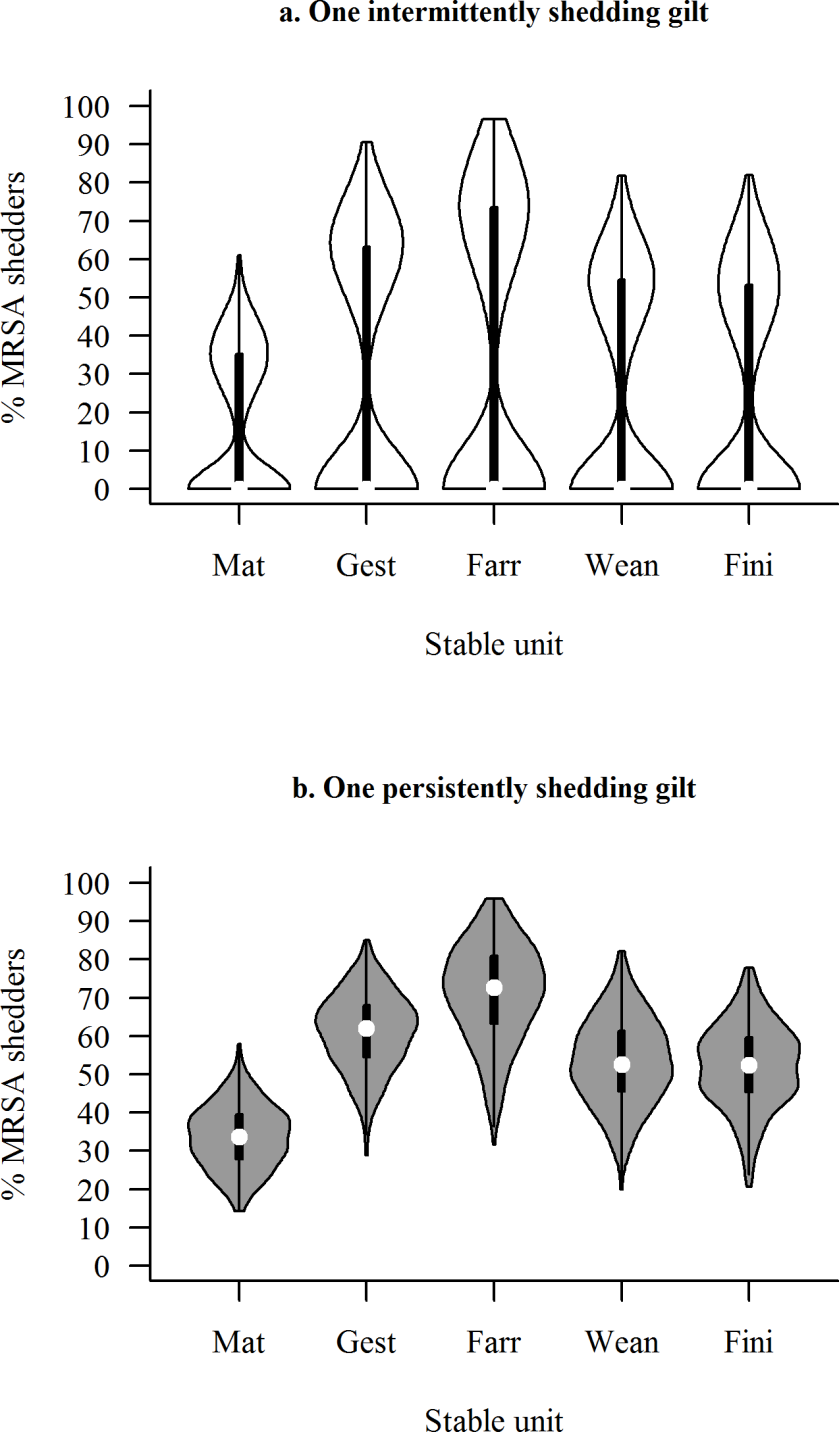
Violin plot of the prevalence following introduction of one gilt shedding MRSA intermittently or persistently. Predicted prevalence of MRSA shedders six years after introduction, when medium transmission rates were used (distribution of 500 iterations). The median prevalences are indicated by white dots. Mat = Mating unit, Gest = Gestation unit, Farr = Farrowing unit, Wean = Weaner unit, Fini = Finisher unit.

Following introduction of a PS instead of an IS into either stable unit, similar developments in median prevalence of MRSA shedders over time were predicted, except that in most cases MRSA was not predicted to fade out, when low or medium transmission rates were used (Fig 3D). The proportion of MRSA shedders in the five different stable units, six years after introduction of an IS or a PS in the mating unit is illustrated in a violin plot in Fig 4. As seen from the distribution of the prevalences of MRSA shedders obtained in the 500 iterations (the width of the ‘violins’), MRSA seems to either fade out when introduced by an IS, or show a pattern similar to when introduced by a PS, where the observed prevalences clustered around the median.

The model predicts that when spread of MRSA kicks off, the predicted prevalence of MRSA shedders within each stable unit reaches an equilibrium (Fig 4). As expected, the age group in which MRSA was introduced had no marked influence on the equilibrium prevalence (S5 and S6 Figs).

Table 2 shows the total median proportion of MRSA shedders in the herd six years after various introductions, as well as the proportion of iterations, where MRSA faded out, including the number of days elapsed between introduction and fade-out. In general the higher transmission rate used the higher prevalence after stabilisation and the lower proportion of iterations, where MRSA fades out (Table 2). When the lower sets of transmission rates was applied, MRSA was predicted to be able to remain in the herd for years, and still eventually fade out. In theory, MRSA fade-out following introduction by a PS is in most cases only possible after the initial PS has been removed from the farm, and therefore MRSA could remain in the herd for a long time despite the infection not becoming established, if it was introduced by an animal with a long lifespan, e.g. a gilt (Table 2).

**Table 2:** Predicted prevalence and fade-out of MRSA in a simulated pig herd following single introductions.

Transmission rates	Introduction scenario	Shedder prevalence		Fade out	Duration	
		Median	5th-95th percentile	(% iterations)	Median	Range
Low	1 IS gilt	0.0	0–38.0	87.0	13.0	1–142
	1 PS gilt	0.0	0–0.01	88.4	507.0	469–557
	1 IS weaner	0.0	0–0	99.2	14.5	1–257
	1 PS weaner	0.0	0–0	95.4	150.0	11–658
	1 IS finisher	0.0	0–0	99.6	14.0	2–312
	1 PS finisher	0.0	0–0	98.4	94.0	1–425
Medium	1 IS gilt	0.0	0–68.6	51.0	13.0	2–100
	1 PS gilt	56.4	39.4–70.2	0.0	-	-
	1 IS weaner	43.9	0–69.0	46.0	11.0	2–347
	1 PS weaner	56.1	0–69.7	7.0	150.0	3–346
	1 IS finisher	0.0	0–68.5	58.4	15.0	1–314
	1 PS finisher	54.1	0.68.9	27.4	100.0	80–444
High	1 IS gilt	64.7	0–79.6	26.4	9.0	2–80
	1 PS gilt	67.0	48.2–79.4	0.0	-	-
	1 IS weaner	64.6	0–82.3	20.6	7.0	1–153
	1 PS weaner	68.0	54.5–80.3	0.4	100.5	9–192
	1 IS finisher	64.7	0–80.6	28.4	8.0	1–128
	1 PS finisher	67.8	51.1–78.8	0.0	-	-

The introduction of more animals increased the probability of MRSA becoming established on the farm (S7 Fig and S10 Table), and shorter time passed before an equilibrium was reached (S8 Fig). However, already with the introduction of thirty instead of ten finishers, the time needed for the MRSA prevalence to stabilize was very similar (S8 Fig). When comparing single or multiple introductions, exemplified by the introduction of one, three or ten IS gilts either once or once every fortnight for three months, the patterns predicted were very similar, since the only major difference was an increased probability of fade-out following single introductions, in particular for one shedder only (S9 and S10 Figs and S10 Table).

### Sensitivity and robustness analysis

**Fig 5:**
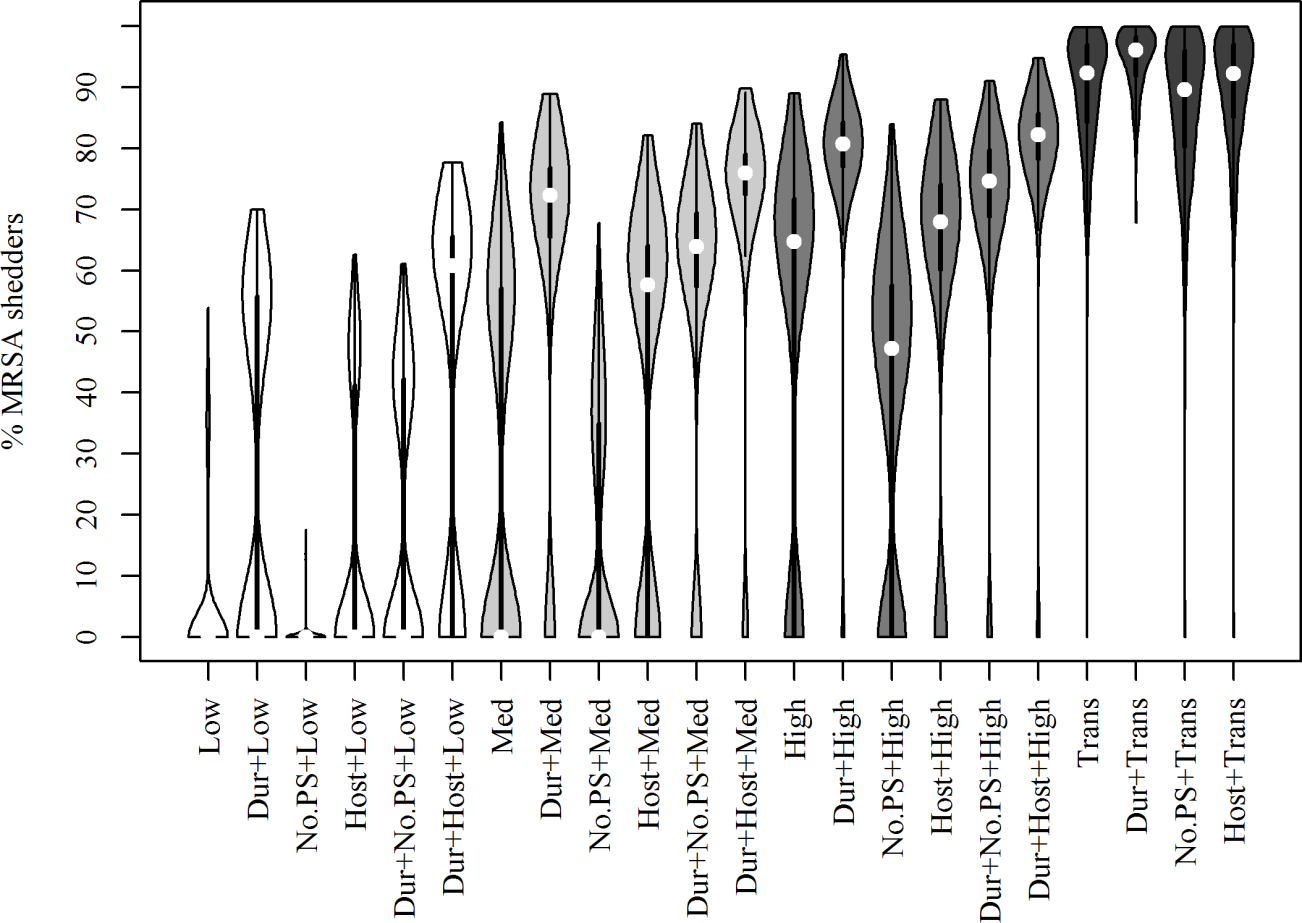
Results of sensitivity- and robustness analysis. Predicted prevalence six years after introduction of one intermittently shedding gilt (distribution of 500 iterations). Last part of each label indicates the transmission rate used. Dur = duration of shedding for IS altered, No.PS = no persistent shedders, Host = shedder type solely determined by host factors (no influence of prevalence in the room), Trans = transmission rates altered.

When modelling introduction of one intermittently shedding gilt with different alternative parameterisations, increasing the duration of shedding led to an increased median prevalence, less variance and fewer iterations, where MRSA faded out (Fig 5 and S8 Table). Removing the possibility of any pigs becoming PS led to MRSA more frequently fading out. Modelling persistent carriage as only being dependent on host-related factors did lead to less cases, where MRSA faded out, compared to using the original distribution. The alternative set of higher transmission rates introduced in the robustness analysis for all age groups predicted higher median prevalence and less variation between the results of the different iterations, except when there was no persistent shedders.

## Discussion

In the present study, we modelled the spread of MRSA between animals within a pig farm mechanistically. The observed effects of different simulated introductions were in line with what one would expect a priori. Our results show that once MRSA has become established in a herd, it will maintain a prevalence that varied depending on factors such as the pig unit and transmission rate, e.g. the median prevalence reaching up to 76% following introduction of one IS gilt when high transmission is assumed (Fig 3C). The variation in the within-herd prevalence in different age groups has been reported before ([2] and S11 Table). Many studies have reported an increase in MRSA prevalence after weaning, followed by a decline in the prevalence before slaughter age (S11 Table), but as expected there was variation and others did not observe any significant difference [34]. In a Swiss study, where individual pigs were followed over time, the highest proportion of pigs changing status from negative to positive were observed when piglets were from 1–14 days old, where the highest proportion of pigs changing status from positive to negative were observed in the last part of the finisher period (between 15–19 weeks and 21–25 weeks of age) [35]. Due to the parameterisation of our model, the prevalence was generally predicted to be highest in piglets in the farrowing unit, before decreasing in the weaner and finisher units, where it persisted at similar levels, albeit slightly lower in the finisher unit. This can be changed though and the model can relatively easily be calibrated to other prevalence levels within the different units by for instance adjusting the transmission rates, which the model is already prepared for. For the purpose of the current study, calibration for specific situations is not necessary. Nevertheless, this may be important when studying the impact of interventions to control MRSA within the herd and the success of these interventions given different MRSA within-herd prevalences.

Introduction of more MRSA shedders and multiple introductions led to faster spread. Despite the assumption of no use of risk antimicrobials (tetracyclines and β-lactam antibiotics) in the herd (and therefore use of the low transmission rates associated with this), introduction of MRSA shedders in a few cases (0.6–13.0%) still led MRSA to spread throughout the herd and become established. Thus a low antimicrobial usage within a herd may not always be sufficient to prevent MRSA from spreading and becoming established, once it has been introduced.

The observation of MRSA being able to fade out following introduction of a few IS, does not seem unrealistic given that during an investigation on Norwegian pig farms, 32 of 51 farms did not become MRSA positive, despite having positive suppliers [36]. For twelve farms, this was explained by the farms only being sporadically supplied from the infected farms, which is therefore comparable to the scenarios modelled.

In our model predictions, MRSA spreads relatively easy between the different units of the farm. MRSA is mainly spread forward in the production chain through movement of pigs, but spread to all units was also predicted when MRSA was introduced in the weaner or finisher section. This was a consequence of our assumptions, since to our best knowledge no between-compartment transmission rates for MRSA on pig farms have been published. Therefore we assumed the between-section and between-unit transmission rates to be a smaller fraction of the between-pen transmission rate, 0.15–0.20 and 0.02 respectively. The true risk of transmission will dependent on multiple local factors i.e. internal biosecurity and design and location of stable units in relation to each other. However, given detection of MRSA in substantial levels in the air inside and outside pig barns [16,37] and the risk of carry-over with workers and equipment, we find it justified to assume that this spread could occur.

The transmission rates used were based on data from a study carried out at four Dutch farms [25]. As for all other studies based on a limited amount of animals, prudence is needed, when interpreting the results. Differences between farms regarding management, antimicrobial use and stable design will potentially influence the transmission rates. This can be reflected in the model by for instance adjusting the transmission rates to reflect different prevelance situations as discussed above.

During 2007–2012 the overall use of antimicrobials for farm animals in the Netherlands was reduced with 56% [38]. However in 2010, the year after the study the transmission rates originates from was conducted, the total antimicrobial use for pigs in the Netherlands was 35% higher than in Denmark [39]. With regard to the groups of drugs considered risk antimicrobials [25], especially the use of tetracyclines for pigs was markedly higher in the Netherlands, whereas some narrow spectrum penicillins were used more in Denmark [39]. On the other hand, in Denmark weaners may get prescribed zinc supplementation in the feed, whereas this is not allowed in the Netherlands [40]. This practise might also influence nasal carriage of MRSA, since there seems to be a genetic linkage between *mecA* and *czrC*, which is coding for zinc resistance [14,41,42]. Thus using transmission rates based on no use of zinc, as is the case in our model, might lead to an underestimation of the transmission frequency in the weaning units [43], whereas using transmission rates based on higher antimicrobial consumption might lead to overestimation.

In our model we used transmission rates based on naturally contaminated pigs housed in ordinary farms. Another approach would have been to rely on data from a transmission study, where pigs housed in animal experimental facilities had been inoculated with MRSA [33]. These transmission rates are considerably higher than the rates used in our model. However, despite the risk of underestimating the true rate of transmission, we believe that for our purpose, it will be more appropriate to use data from naturally contaminated pigs housed in an ordinary farm environment, since management practices, animal density and environmental spread play an important role in transmission [44].

The association between the MRSA status of the sow and the probability of piglets testing MRSA positive have been confirmed in several studies [45–47]. In our model, the probability of transmission from sow to new-born piglets was based on predictions from a study, where piglets had been sampled within one hour after birth and again after one day [47]. It has been suggested that piglets might get transiently rather than persistently colonized from their dam [45]. However this has not been taken into account in the model, meaning that the proportion of piglets becoming PS might be overestimated.

After considering different model structures, we chose to assume that IS and PS constituted two distinct groups in pigs, based on evidence in humans [28] and potential evidence in pigs [26].

The proportion of pigs assumed to have the potential to become PS was based on a study at 20 Danish pig farms, where the proportion of pigs persistently testing positive for *S*. *aureus* was 24% [26]. In a study conducted at four Belgian farms, no PS was found at two mildly contaminated farms (17–33% IS), while 25–92% of sows at two highly contaminated farms did persistently test MRSA positive (all the remaining sows at these two farms were IS) [48]. Therefore it seems reasonable that we introduce a prevalence dependency in the model, and thereby take the effect of the contamination level into account.

Results of the sensitivity analysis demonstrated that increasing the duration of carriage led to equilibrium occurring at a markedly higher prevalence compared to the default values (Fig 5 and S8 Table). This was also the case in the robustness analysis, when increased duration was combined with parameter changes that otherwise were expected to decrease the equilibrium prevalence. Altogether, these results indicate that duration of carriage has a considerable influence on the results obtained. This duration may potentially be influenced by many different factors, such as dose of exposure, genetics and the nasal microbiome of the pig [26,49–52]. Removing persistent shedders in the sensitivity analysis interestingly also had a markedly effect, which indicates that there might be some potential in control options targeted at this particular subgroup of animals. As expected, increasing the transmission rate had a pronounced effect and resulted in higher equilibrium prevalences and in some cases, the predictions reached 100% (Fig 5 and S8 Table).

As for all simulation models, the precision, uncertainty and validity of the model predictions will depend on the availability and quality of data for parameterisation of the model and the assumptions and simplifications made. Therefore prudence is called for when interpreting model predictions, which only should be taken as indicative of how MRSA might spread. Despite these limitations, our simulation model can assist in: highlighting knowledge gaps for future research; providing insights in the dynamics of spread of MRSA; the study of possible hypothetical scenarios; and investigation of possible intervention strategies or surveillance options.

## Supporting information

S1 Appendix Estimation of transmission rates. (PDF)

S1 Table Model input: Probabilities of sow parities at simulation start and re-insemination attempts. (PDF)

S2 Table Model input: Litter size, duration of shedding and transmission rates used for sensitivity analysis. (PDF)

S3 Table Model input: Probability of removal of sows. (PDF)

S4 Table Model input: Assumed slaughter age distribution. (PDF)

S5 Table Model input: Removal of piglets, weaners and finishers. (PDF)

S6 Table Model input: Probability of pigs becoming persistent shedders. (PDF)

S7 Table Model input: Transmission rates and probabilities. (PDF)

S8 Table Model output: Results of sensitivity- and robustness analysis. (PDF)

S9 Table Model output: Simulated production parameters compared to Danish production data. (PDF)

S10 Table Model output: Predicted fade out of MRSA in a simulated pig herd and time elapsed between introduction and fade out following single or multiple introductions. (PDF)

S11 Table Summary of MRSA prevalence in different age groups in observational studies. (PDF)

S1 Fig The sow cycle modelled in a hypothetical farrow-to-finish herd. (PDF)

S2 Fig Model output: Convergence after introduction of one intermittently shedding gilt. (PDF)

S3 Fig Model output: Development in the median prevalence of MRSA shedders following introduction of one MRSA shedding weaner. (PDF)

S4 Fig Model output: Development in the median prevalence of MRSA shedders following introduction of one MRSA shedding finisher. (PDF)

S5 Fig Model output: Violin plot of the prevalence following introduction of one weaner shedding MRSA intermittently or persistently. (PDF)

S6 Fig Model output: Violin plot of the prevalence following introduction of one finisher shedding MRSA intermittently or persistently. (PDF)

S7 Fig Model output: Violin plot of the prevalence following introduction of one, ten or thirty finishers shedding MRSA intermittently. (PDF)

S8 Fig Model output: Development in the median prevalence of MRSA shedders following introduction of one, ten or thirty IS finishers. (PDF)

S9 Fig Model output: Violin plot of the prevalence following introduction of one, three or ten gilt shedding MRSA intermittently every fortnight for three months. (PDF)

S10 Fig Model output: Development in the median prevalence of MRSA shedders following single or multiple introductions. (PDF)
