# Supplementary material for: A mechanistic model for spread of livestock-associated methicillin-resistant Staphylococcus aureus (LA-MRSA) within a pig herd
Source: PLoS One. 2017 Nov 28;12(11):e0188429. doi: 10.1371/journal.pone.0188429 (PMC5705068; doi:10.1371/journal.pone.0188429)
Supplement: S1 Appendix — (PDF) [file pone.0188429.s001.pdf]

S1 Appendix: Estimation of transmission rates

The transmission rates (β) used in our simulation model was drawn from a pert distribution.

The values used as most likely, min. and max. value in the distribution was calculated from the reproduction ratio (R_0) and corresponding the 95% CI reported by Broens et al., 2012a, combined with the duration of carriage (D) used in the same study (estimated by Broens et al., 2012b) as:

$$\beta = R_0/D, \quad \text{(A1)}$$

where D was 17.4 days.

Broens et al., 2012a introduces the term total infection pressure (IP) defined as:

$$IP = IP \text{ within the pen} + IP \text{ other pens} + IP \text{ environment}, \quad \text{(A2)}$$

where IP within the pen = proportion of infectious pigs within the pen, IP other pens = proportion of infectious pen within the compartment, but not in the same pen, and IP environment = proportion of positive environmental wipes.

Based on, this Broens et al., 2012a also introduced a variable (pIP) to describe the relative effect of transmission through direct contact with pen mates:

$$pIP = IP \text{ within the pen} / IP \quad \text{(A3)}$$

R_0 reported by Broens et al., 2012a, when pIP=1 was used for estimation of within-pen transmission rates, whereas between- pen transmission rates were estimated based on R_0 when pIP=0.

References

Broens, E.M., Espinosa-Gongora, C., Graat, E.A.M., Vendrig, N., Van Der Wolf, P.J., Guardabassi, L., Butaye, P., Nielsen, J.P., De Jong, M.C.M., Van De Giessen, A.W., 2012a. Longitudinal study on transmission of MRSA CC398 within pig herds. BMC Vet. Res. 8, 58. doi:10.1186/1746-6148-8-58

Broens, E.M., Graat, E. a M., van de Giessen, A.W., Broekhuizen-Stins, M.J., de Jong, M.C.M., 2012b. Quantification of transmission of livestock-associated methicillin resistant Staphylococcus aureus in pigs. Vet. Microbiol. 155, 381–388. doi:10.1016/j.vetmic.2011.09.010
