# Supplementary material for: A mechanistic model for spread of livestock-associated methicillin-resistant Staphylococcus aureus (LA-MRSA) within a pig herd
Source: PLoS One. 2017 Nov 28;12(11):e0188429. doi: 10.1371/journal.pone.0188429 (PMC5705068; doi:10.1371/journal.pone.0188429)
Supplement: S1 Table — (PDF) [file pone.0188429.s002.pdf]

Table S1: Model input: Sow parities at simulation start and re-insemination attempts

**Parity**	**1**	**2**	**3**	**4**	**5**	**6**	**7**	**8**
Probability matrix for sow parities at start of simulation	0.22	0.20	0.17	0.14	0.11	0.09	0.05	0.02
Probability of re-insemination being attempted*	1.0	0.7	0.6	0.5	0.4	0.3	0.2	0.0

*: Sørensen G, Christiansen MG. Udsætningsstrategi (in Danish). Danish Research Centre for pigs; 2013. pp. 1–8. Available: http://vsp.lf.dk/Viden/Reproduktion/Udsaetningsstrategi.aspx
