# Supplementary material for: A mechanistic model for spread of livestock-associated methicillin-resistant Staphylococcus aureus (LA-MRSA) within a pig herd
Source: PLoS One. 2017 Nov 28;12(11):e0188429. doi: 10.1371/journal.pone.0188429 (PMC5705068; doi:10.1371/journal.pone.0188429)
Supplement: S2 Table — (PDF) [file pone.0188429.s003.pdf]

S2 Table. Model input: Litter size, duration of shedding and transmission rates used for sensitivity analysis

Parameter	Distribution	Mean	SD	Most likely	Min	Max
Litter size[1]	Normal	15.9	1.5	-	-	-
Shedding duration (days)[2]	Pert	-	-	7.5	1	26
Alt. Shedding duration (days	Pert	-	-	18	6	29
Alt. BetaWP[3]	Pert	-	-	1.57	0.86	2.92
Alt. BetaBP[4]	Pert	-	-	0.31	0.17	0.57

1: Jessen, 2016 [1]. The SD is an assumption. The numbers drawn were rounded to integers.

2: Broens, 2012a [2].

3: Within pen transmision rates (Alt. BetaWP) : Crombé et al., 2012 [3].

4: Calculated from Crombé et al, 2012 and BetaWP/BetaBP ratio in Broens et al, 2012b [3,4].

References

1. Jessen O. Landsgennemsnit for produktivitet i svineproduktionen 2015 (in Danish) [Internet]. 2016. Available: vsp.lf.dk/~/media/Files/PDF - Publikationer/.../Notat_1611.pdf

2. Broens EM, Graat EAM, van de Giessen AW, Broekhuizen-Stins MJ, de Jong MCM. Quantification of transmission of livestock-associated methicillin resistant *Staphylococcus aureus* in pigs. Vet Microbiol. Elsevier B.V.; 2012a;155: 381–388. doi:10.1016/j.vetmic.2011.09.010

3. Crombé F, Vanderhaeghen W, Dewulf J, Hermans K, Haesebrouck F, Butaye P. Colonization and transmission of methicillin-resistant *Staphylococcus aureus* ST398 in nursery piglets. Appl Environ Microbiol. 2012;78: 1631–1634. doi:10.1128/AEM.07356-11

4. Broens EM, Espinosa-Gongora C, Graat EAM, Vendrig N, Van Der Wolf PJ, Guardabassi L, et al. Longitudinal study on transmission of MRSA CC398 within pig herds. BMC Vet Res. 2012b;8: 58. doi:10.1186/1746-6148-8-58
