# Supplementary material for: A mechanistic model for spread of livestock-associated methicillin-resistant Staphylococcus aureus (LA-MRSA) within a pig herd
Source: PLoS One. 2017 Nov 28;12(11):e0188429. doi: 10.1371/journal.pone.0188429 (PMC5705068; doi:10.1371/journal.pone.0188429)
Supplement: S3 Table — (PDF) [file pone.0188429.s004.pdf]

S3 Table. Model input: Probability of removal of sows

Parity	1	2	3	4	5	6	7	8
Daily probability of removal between insemination and farrowing[1]	0.00018	0.00014	0.00015	0.00024	0.00016	0.0002	0.00018	0.0003
Daily probability of removal after farrowing and before weaning[2]	0.00085	0.00079	0.00094	0.00103	0.00094	0.00076	0.00127	0.00085
Probability of removal immediately after weaning[3]	0.040	0.024	0.026	0.050	0.108	0.256	0.354	1.000

All probabilities were calculated based on data from Sørensen & Christiansen, 2013 and assumptions regarding duration of the different stages in the sow cycle.

1: The total probability for the whole period ranged from 0.016 to 0.034

2: The total probability for the whole period ranged from 0.025 to 0.034

3: The probability of removal immediately after weaning was adjusted for removal of sows experiencing insemination failure, where re-insemination was not attempted (according to the probabilities in table S1)

References

Sørensen G, Christiansen MG. Udsætningsstrategi (in Danish). Danish Research Centre for pigs; 2013. pp. 1–8. Available: http://vsp.lf.dk/Viden/Reproduktion/Udsaetningsstrategi.aspx
