# Supplementary material for: A mechanistic model for spread of livestock-associated methicillin-resistant Staphylococcus aureus (LA-MRSA) within a pig herd
Source: PLoS One. 2017 Nov 28;12(11):e0188429. doi: 10.1371/journal.pone.0188429 (PMC5705068; doi:10.1371/journal.pone.0188429)
Supplement: S4 Table — (PDF) [file pone.0188429.s005.pdf]

S4 Table. Model input: Assumed slaughter age distribution

Age (days)	% pigs per batch
151	2
158	8
165	80
172	8
179	2
