# Supplementary material for: A mechanistic model for spread of livestock-associated methicillin-resistant Staphylococcus aureus (LA-MRSA) within a pig herd
Source: PLoS One. 2017 Nov 28;12(11):e0188429. doi: 10.1371/journal.pone.0188429 (PMC5705068; doi:10.1371/journal.pone.0188429)
Supplement: S5 Table — (PDF) [file pone.0188429.s006.pdf]

S5 Table. Model input: Removal of piglets, weaners and finishers

	Total probability	Daily probability	
Piglets[1]	0.13	Day 1	0.03752
		Day 2	0.02010
		Day 3	0.01474
		Day 4-7	0.00670
		Day 8-14	0.00322
		Day 15-28	0.00094
Weaners[2]	0.03		0.00060
Finishers[2]	0.06		0.00007

1: Assumptions for daily probabilities of removal are based on [1].

2: Total probability based on [2]. Finisher mortality was calculated as total mortality in finisher herds minus mortality in weaner herds.

References

1. Pedersen LJ, Berg P, Jørgensen E, Bonde MK, Herskin MS, Knage-rasmussen KM, et al. Pattegrisdødelighed i DK (in Danish). 2010. Available: http://web.agrsci.dk/djfpublikation/djfpdf/Rapport_86_husdyrbrug_53458_samlet.pdf

2. Jessen O. Landsgennemsnit for produktivitet i svineproduktionen 2015 (in Danish). 2016. Available: vsp.lf.dk/~/media/Files/PDF - Publikationer/.../Notat_1611.pdf
