# Supplementary material for: A mechanistic model for spread of livestock-associated methicillin-resistant Staphylococcus aureus (LA-MRSA) within a pig herd
Source: PLoS One. 2017 Nov 28;12(11):e0188429. doi: 10.1371/journal.pone.0188429 (PMC5705068; doi:10.1371/journal.pone.0188429)
Supplement: S6 Table — (PDF) [file pone.0188429.s007.pdf]

S6 Table. Model input: Probability of pigs becoming persistent shedders

Parameter	Distribution	Mean/ most likely value	Min	Max/SD
Probability of having the potential to become a persistent shedder	Normal	0.24	-	0.01
Prevalence threshold	Pert	0.70	0.50	1.00
Probability of persistent shedding below threshold	Pert	0.1	0.01	0.40
Probability of persistent shedding above threshold	Pert	0.75	0.50	1.00

Note: All parameters were based on assumptions inspired by Espinosa-Gongora et al., 2015.

Probability of having the potential to become a persistent shedder: The probability of an individual pig being assigned the potential to become a persistent carrier, provided it will be exposed to sufficiently high levels of MRSA.

Prevalence threshold: The prevalence cut-off value for when higher or lower probability for potential persistent shedders becoming persistent shedders should be applied.

Probability of persistent shedding below threshold: The probability of potential persistent shedders becoming persistent carriers, if the prevalence within the room, where they are housed is below or equal to the PrevCutOff.

Probability of persistent shedding above threshold = Probability of potential persistent shedders becoming persistent carriers, if the prevalence within the room, where they are housed is above PrevCutOff.

References

Espinosa-Gongora C, Dahl J, Elvstrøm A, van Wamel WJ, Guardabassi L. Individual predisposion to *Staphylococcus aureus* colonization in pigs on the basis of quantification, carriage dynamics, and serological profiles. Appl Environ Microbiol. 2015;81: 1251–6. doi:10.1128/AEM.03392-14
