# Supplementary material for: A mechanistic model for spread of livestock-associated methicillin-resistant Staphylococcus aureus (LA-MRSA) within a pig herd
Source: PLoS One. 2017 Nov 28;12(11):e0188429. doi: 10.1371/journal.pone.0188429 (PMC5705068; doi:10.1371/journal.pone.0188429)
Supplement: S7 Table — (PDF) [file pone.0188429.s008.pdf]

S7 Table. Model input: Transmission rates and probabilities

Route	Agegroup	Most likely value[1]	Min.[2]	Max.[2]	Source/calculated from
**Transmission rates**					
Within pen (Low)	Weaner	0.0701	0.0345	0.1425	Broens et al., 2012
	Finisher	0.0701	0.0345	0.1425	Broens et al., 2012
	Others	0.0701	0.0345	0.1425	Broens et al., 2012
	Sow→offspring	0.1816	0.0655	0.5069	Broens et al., 2016
	Piglets	0.1816	0.0655	0.5069	Broens et al., 2016
Between pens (Low)	Weaner	0.0138	0.0103	0.0178	Broens et al., 2012
	Finisher	0.0138	0.0103	0.0178	Broens et al., 2012
	Others	0.0138	0.0103	0.0178	Broens et al., 2012
	Mixed	0.0351	0.0195	0.0632	Broens et al., 2012
Within pen (Med)	Weaner	0.1247	0.0503	0.3144	Average of low and high
	Finisher	0.1247	0.0503	0.3144	Average of low and high
	Others	0.1247	0.0503	0.3144	Average of low and high
	Sow→offspring	0.3230	0.0951	1.1190	Average of low and high
	Piglets	0.3230	0.0951	1.1190	Average of low and high
Between pens (Med)	Weaner	0.0241	0.0149	0.0394	Average of low and high
	Finisher	0.0241	0.0149	0.0394	Average of low and high
	Others	0.0241	0.0149	0.0394	Average of low and high
	Mixed	0.0624	0.0284	0.1399	Average of low and high
Within pen (High)	Weaner	0.1793	0.0661	0.4862	Broens et al., 2012
	Finisher	0.1793	0.0661	0.4862	Broens et al., 2012
	Others	0.1793	0.0661	0.4862	Broens et al., 2012
	Sow→offspring	0.4644	0.1247	1.7310	Broens et al., 2016
	Piglets	0.4644	0.1247	1.7310	Broens et al., 2016
Between pens (High)	Weaner	0.0345	0.0195	0.0609	Broens et al., 2012
	Finisher	0.0345	0.0195	0.0609	Broens et al., 2012
	Others	0.0345	0.0195	0.0609	Broens et al., 2012
	Mixed	0.0897	0.0374	0.2167	Broens et al., 2012
Between sections	Farrowing sec.	0.20*BetaBPO			Assumption
	All other sec.	0.15*BetaBPO			Assumption
Between stables	All	0.02*BetaBPO			Assumption
**Probabilities**					
Sow to piglets	Newborns (pos. dam)	0.75	0.56	0.91	Verhegghe et al., 2013
(day 1)	Newborns (neg. dam)[3]	0.35	0.26	0.46	Verhegghe et al., 2013

1: All values for transmission rates are calculated from R0 values, whereas the probabilities are mean predictions read from a figure.

2: The lower and upper limits of 95% confidence intervals for the most likely value were used as min. and max. values in the pert distribution.

3: Probability of infection as newborn (during the first day of life) given that the piglet is born by an uninfected sow, but in a unit with infected animals. If there are no infected animals within the section, this value will be zero

References

Broens EM, Espinosa-Gongora C, Graat EAM, Vendrig N, Van Der Wolf PJ, Guardabassi L, et al. Longitudinal study on transmission of MRSA CC398 within pig herds. BMC Vet Res. 2012b;8: 58. doi:10.1186/1746-6148-8-58
