# Supplementary material for: A mechanistic model for spread of livestock-associated methicillin-resistant Staphylococcus aureus (LA-MRSA) within a pig herd
Source: PLoS One. 2017 Nov 28;12(11):e0188429. doi: 10.1371/journal.pone.0188429 (PMC5705068; doi:10.1371/journal.pone.0188429)
Supplement: S8 Table — (PDF) [file pone.0188429.s009.pdf]

S8 Table: Results of sensitivity- and robustness analysis

Parameterisation (Transmission rate + modification)	Shedder prevalence		Fade out (% iterations)	Days before fade-out	
	Median	5th-95th percentile		Median	Range
Low	0.0	0-38.0	87.0	13.0	1-142
Med	0.0	0-68.6	51.0	13.0	2-100
High	64.7	0-79.6	26.4	9.0	2-80
Low+Dur	0.0	0-63.5	51.8	27	7-268
Med+Dur	72.3	0-83.4	17.0	24	8-81
High+Dur	80.7	69.6-89.2	3.8	21	7-45
Low+No.PS	0.0	0-0	98.4	14	2-1902
Med+No.PS	0.0	0-51.5	58.2	16	1-108
High+No.PS	47.2	0-70.6	28.8	12	2-199
Low+Host	0.0	0-52.8	72.4	11	2-248
Med+Host	57.7	0-72.3	34.4	9	1-34
High+Host	67.9	0-81.4	19.0	8	1-38
Low+Dur+No.PS	0.0	0-51.9	57.6	23	7-590
Med+Dur+No.PS	63.9	0-77.3	15.0	21	10-78
High+Dur+No.PS	74.6	0-85.6	6.4	18.5	10-52
Low+Dur+Host	60.8	0-71.4	37.6	21	7-139
Med+Dur+Host	76.0	0-83.7	9.6	16.5	7-60
High+Dur+Host	82.2	68.2-90.1	4.6	18	6-43
Trans	92.4	71.5-99.5	0.2	10	-
Trans+Dur	96.0	84.8-99.7	0.0	-	-
Trans+No.PS	89.6	65.5-99.4	0.4	4.5	4-5
Trans+Host	92.3	71.5-99.4	0.2	4	-

Dur = duration of shedding for IS altered,
No.PS = no persistent shedders,
Host = shedder type solely determined by host factors (no influence of prevalence in the room),
Trans = low, medium or high transmission rates based on Broens et al., 2012 replaced by rates based on Crombé et al., 2012.

References

Broens EM, Espinosa-Gongora C, Graat EAM, Vendrig N, Van Der Wolf PJ, Guardabassi L, et al. Longitudinal study on transmission of MRSA CC398 within pig herds. BMC Vet Res. 2012;8: 58. doi:10.1186/1746-6148-8-58

Crombé F, Vanderhaeghen W, Dewulf J, Hermans K, Haesebrouck F, Butaye P. Colonization and transmission of methicillin-resistant *Staphylococcus aureus* ST398 in nursery piglets. Appl Environ Microbiol. 2012;78: 1631–1634. doi:10.1128/AEM.07356-11
