# Supplementary material for: A mechanistic model for spread of livestock-associated methicillin-resistant Staphylococcus aureus (LA-MRSA) within a pig herd
Source: PLoS One. 2017 Nov 28;12(11):e0188429. doi: 10.1371/journal.pone.0188429 (PMC5705068; doi:10.1371/journal.pone.0188429)
Supplement: S9 Table — (PDF) [file pone.0188429.s010.pdf]

S9 Table: Model output: Simulated parameters compared to Danish production data.

Parameter	Model performance[1]	DK production 2015[2]
No. of liveborn piglets per litter	15.3	15.9
No. of weaned piglets per litter	13.5	13.8
Lactation time, days	28*[,D]	30
Dead before weaning, %	13.2	13.4
Litters from first parity sows,%	20.0	23.5
From weaning to insemination, days	6.0[D]	5.7
Farrowing rate, %	87.5	88.1
Re-inseminated, %	6.2	5.3
Slaughterpigs, dead or rejected at the abattoir, %	3.7[D]	3.7

* Nursery sows not included.

D: Defined as this value.

1: Medians of medians per iteration based on 6 years run with 500 iterations (after 3 years pre-run).

2: Jessen, O. / VSP (2016): Notat 1611: Landsgennemsnit for produktivitet i svineproduktionen, 2015.
