# Supplementary material for: A mechanistic model for spread of livestock-associated methicillin-resistant Staphylococcus aureus (LA-MRSA) within a pig herd
Source: PLoS One. 2017 Nov 28;12(11):e0188429. doi: 10.1371/journal.pone.0188429 (PMC5705068; doi:10.1371/journal.pone.0188429)
Supplement: S10 Table — (PDF) [file pone.0188429.s011.pdf]

S10 Table. Model output: Predicted fade out of MRSA in a simulated pig herd and time elapsed between introduction and fade out following single or multiple introductions

Transmission rates	Introduction scenario	Shedder prevalence		Fade out (% iterations)	Duration (days)	
		Median	5th-95th percentile		Median	Range
Low	3 IS gilts	0.0	0-39.1	79.6	21	2-304
	3 PS gilts	31.2	16.1-45.9	0.2	1248	-
	10 IS gilts	17.7	0-44.3	48.2	31	3-322
	10 PS gilts	32.0	17.1-45.7	0.0	-	-
	10 IS weaner	0.0	0-31.5	92.8	142	2-669
	10 PS weaner	0.0	0-44.2	75.8	192	142-584
	10 IS finisher	0.0	0-0	97.8	86	2-528
	10 PS finisher	0.0	0-40.1	87.2	129	94-758
	30 IS finisher	0.0	0-35.3	92.8	96	7-604
	50 IS finishers	0.0	0-32.9	93.8	100	15-744
	3 IS gilts/14 days	27.5	0-45.8	29.8	94	54-537
	10 IS gilts/14 days	31.0	0-46.0	9.4	106	77-448
	50 IS weaners/14 days	0.0	0-44.6	51.2	268	170-1040
	100 IS weaners/14 days	27.1	0-48.3	39.0	276	156-758
	50 IS finishers/14 days	0.0	0-45.6	65.2	206	100-528
	100 IS finishers/14 days	0.0	0-44.5	54.0	226	130-640
Medium	3 IS gilts	53.2	0-67.8	23.6	23	1-178
	3 PS gilts	56.5	39.9-72.0	0	-	-
	10 IS gilts	55.3	0-69.2	8.0	33	5-106
	10 PS gilts	56.3	40.4-68.4	0	-	-
	10 IS weaner	56.8	36.0-69.8	4.2	133	9-394
	10 PS weaner	56.7	41.4-70.6	0.0	-	-
	10 IS finisher	55.1	0-70.4	18.2	94	7-408
	10 PS finisher	56.2	0-70.3	5.4	156	100-410
	30 IS finisher	56.2	0-71.1	10.8	111	21-268
	50 IS finishers	56.9	34.8-69.1	4.2	124	36-318
	3 IS gilts/14 days	56.0	39.5-69.7	2	104.5	59-131
	10 IS gilts/14 days	56.2	39.9-70.6	0	-	-
	50 IS weaners/14 days	56.4	42.2-71.9	0.2	436	-
	100 IS weaners/14 days	57.1	41.7-70.1	0.0	-	-
	50 IS finishers/14 days	56.1	39.2-71.2	0.6	254	151-260
	100 IS finishers/14 days	57.0	40.0-69.9	0.0	-	-

The table continues on the next page.

S10 Table, continued from page 1.

Transmission rates	Introduction scenario	Shedder prevalence		Fade out (% iterations)	Duration (days)	
		Median	5th-95th percentile		Median	Range
High	3 IS gilts	67.5	0-80.4	6.0	19.5	2-54
	3 PS gilts	68.3	54.8-59.9	0	-	-
	10 IS gilts	67.5	52.7-59.5	0.6	39	20-49
	10 PS gilts	67.7	53.2-80.3	0	-	-
	10 IS weaner	68.6	53.1-81.5	0	-	-
	10 PS weaner	68.0	52.4-79.9	0	-	-
	10 IS finisher	67.4	51.6-81.6	0.6	134	94-142
	10 PS finisher	67.8	53.7-81.5	0	-	-
	30 IS finisher	67.9	53.3-80.7	0	-	-
	50 IS finishers	67.5	52.1-80.2	0	-	-
	3 IS gilts/14 days	67.0	52.9-80.4	0.2	120	-
	10 IS gilts/14 days	66.4	47.4-80.6	0	-	-
	50 IS weaners/14 days	67.8	53.3-80.8	0	-	-
	100 IS weaners/14 days	68.3	53.2-81.3	0	-	-
	50 IS finishers/14 days	67.3	52.6-81.4	0	-	-
	100 IS finishers/14 days	67.6	53.4-81.9	0	-	-
