# Supplementary material for: A mechanistic model for spread of livestock-associated methicillin-resistant Staphylococcus aureus (LA-MRSA) within a pig herd
Source: PLoS One. 2017 Nov 28;12(11):e0188429. doi: 10.1371/journal.pone.0188429 (PMC5705068; doi:10.1371/journal.pone.0188429)
Supplement: S11 Table — (PDF) [file pone.0188429.s012.pdf]

S11 Table: Summary of MRSA prevalence in different age groups in observational studies

MRSA prevalence in group[1]	Age in days	Broens et al., 2012[2]	Crombé et al., 2012	Dewaele et al., 2011[3]	Khanna et al., 2008	Merialdi et al., 2012	Merialdi et al., 2013[4]	Nathaus et al., 2010	Pletinckx et al., 2013[5]	Verhegghe et al., 2013[6]	Weese et al., 2011
Sows	Unspec.	33[7]/77[8]	26	96/59	-	-	53.3	10.3	1.4-90.7	17-33/50-100	-
Piglets	See notes	>60	41	100/50	20	1.6	53.3	15.5[9]<br>48.3[11]	4.8-100	0-36/>90	1[10]<br>20[11]
Weaners	See notes	-	-	100/100[12]<br>100/100[16]	28	100	56.7<br>60.0[17]	25.9[13]<br>51.7[18]	73.3-100	69-91/>95[14]	34[15]<br>65[13]<br>50[17]<br>42[19]
Finishers	See notes		26	100/-[20]<br>78/-[23]<br>87/-[24]	26[21]	18.3	31.7		51.1-85.7	60-75/75-80[22]	

Notes:

1: Prevalence of positive nasal swabs, if no other sample type is indicated

2: Based on nasal or rectal swabs

3: Results for two farms presented as farm A/farm B

4: Based on environmental swabs

5: Significant differences were reported between pre- and post-weaned piglets, between post-weaned piglets and fattening pigs, between 11-17 weeks old and 18-20 weeks old fattening pigs and between sows and fattening pigs

6: Reported as results for two low- / to high-contaminated farms. Some of the values are only approximate values, since they have been read from a graph (day 165 for the low-contaminated farms + all values from the highly contaminated farms, except the prevalence range for the sows)

7: Before farrowing

8: At the end of weaning

9: Day 1-3

10: Day 1

11: Day 21

12: Day 28-56
13: Day 42
14: Day 52-58
15: Day 28
16: Day 56-84
17: Day 56
18: Day 63
19: Day 72
20: Day 84-112
21: Agegroup defined as ‘grower-finisher’
22: Day 165
23: Day 112-140
24: Day 140-162

References

1. Broens EM, Espinosa-Gongora C, Graat EAM, Vendrig N, Van Der Wolf PJ, Guardabassi L, et al. Longitudinal study on transmission of MRSA CC398 within pig herds. BMC Vet Res. 2012;8: 58. doi:10.1186/1746-6148-8-58

2. Crombé F, Vanderhaeghen W, Dewulf J, Hermans K, Haesebrouck F, Butaye P. Colonization and transmission of methicillin-resistant *Staphylococcus aureus* ST398 in nursery piglets. Appl Environ Microbiol. 2012;78: 1631–1634. doi:10.1128/AEM.07356-11

3. Dewaele I, Messens W, Man I De, Delputte P, Herman L, Butaye P, et al. Sampling, prevalence and characterization of methicillin-resistant *Staphylococcus aureus* on two Belgian pig farms. Vet Sci Dev. 2011;1: 4–8. doi:10.4081/vsd.2011.e1

4. Khanna T, Friendship R, Dewey C, Weese JS. Methicillin resistant Staphylococcus aureus colonization in pigs and pig farmers. Vet Microbiol. 2008;128: 298–303. doi:10.1016/j.vetmic.2007.10.006

5. Merialdi G, Galletti E, Rugna G, Granito G, Franco A, Battisti A, et al. Longitudinal study on MRSA nasal colonization in a farrow to finish pig herd. 4th European Symposium of Porcine Health Management. 2012. p. 1.

6. Merialdi G, Galletti E, Guazzetti S, Rosignoli C, Alborali G, Battisti A, et al. Environmental methicillin-resistant *Staphylococcus aureus* contamination in pig herds in relation to the productive phase and application of cleaning and disinfection. Res Vet Sci. 2013;94: 425–427. doi:10.1016/j.rvsc.2012.10.020

7. Nathaus R, Blaha T, Tegeler R, Meemken D. *Staphylococcus aureus* in zwei Schweine- zuchtbestanden (Intra-herd prevalence and colonisation dynamics of Methicillin-resistant Staphylococcus au reus (MRSA) in two pig breeding herds) (In German with abstract in English). Berl Munch Tierarztl Wochenschr. 2010;1236: 221–228. doi:10.2376/0005-9366-123-221

8. Pletinckx LJ, Verhegghe M, Crombé F, Dewulf J, De Bleecker Y, Rasschaert G, et al. Evidence of possible methicillin-resistant *Staphylococcus aureus* ST398 spread between pigs and other animals and people residing on the same farm. Prev Vet Med. 2013;109: 293–303. doi:10.1016/j.prevetmed.2012.10.019

9. Verhegghe M, Pletinckx LJ, Crombé F, Weyenberg S Van, Haesebrouck F, Butaye P, et al. Cohort study for the presence of livestock-associated MRSA in piglets: Effect of sow status at farrowing and determination of the piglet colonization age. Vet Microbiol. 2013;162: 679–686. doi:10.1016/j.vetmic.2012.09.014

10. Weese JS, Zwambag A, Rosendal T, Reid-Smith R, Friendship R. Longitudinal Investigation of Methicillin-Resistant *Staphylococcus aureus* in Piglets. Zoonoses Public Health. 2011;58: 238–243. doi:10.1111/j.1863-2378.2010.01340.x
