# Supplementary material for: A mechanistic model for spread of livestock-associated methicillin-resistant Staphylococcus aureus (LA-MRSA) within a pig herd
Source: PLoS One. 2017 Nov 28;12(11):e0188429. doi: 10.1371/journal.pone.0188429 (PMC5705068; doi:10.1371/journal.pone.0188429)
Supplement: S1 Fig — (PDF) [file pone.0188429.s013.pdf]

S1 Fig. The sow cycle modelled in a hypothetical farrow-to-finish herd

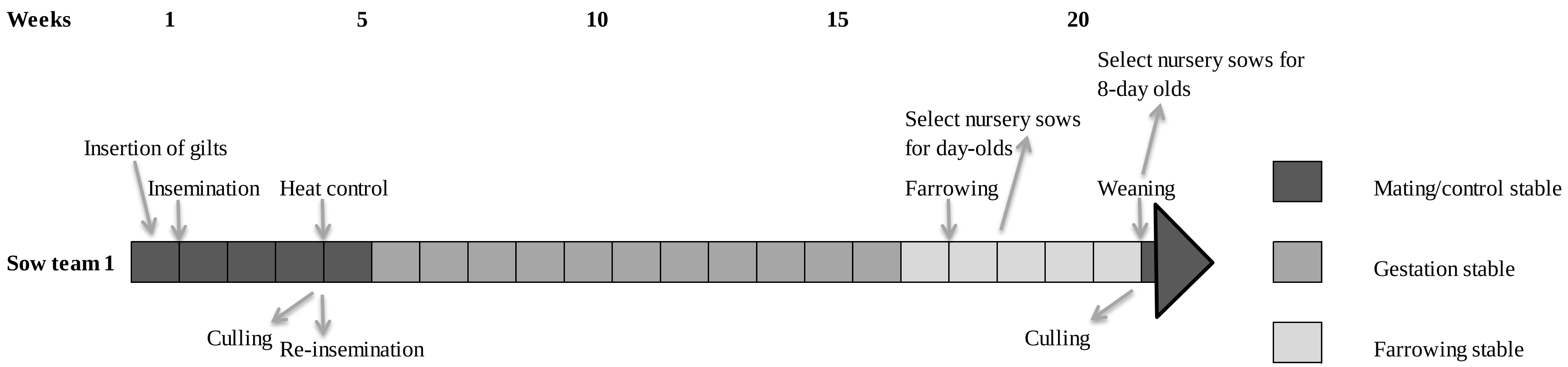

Assumed total duration from insertion in the mating stable to weaning in the farrowing stable = 147 days. It is assumed that sows are inseminated 5 days after arrival in the mating unit. The need for adding gilts to the sow teams are evaluated three days before the insemination. Three weeks after insemination, it is checked if re-insemination is needed for any sows (heat control). It is assumed that some of the pigs needing re-insemination will be culled. The sows remain in the mating and control unit until 4 weeks after insemination. The duration of the gestation period is assumed to be 114 days. Sows are moved to the farrowing unit 5 days before expected farrowing. Piglets will be weaned after 28 days. Strategic culling of sows will take place immediately after weaning (or after insemination failure has been observed). Emergency culling or deaths can occur anywhere in the cycle. Two-step nursery sows are selected among sows who farrowed 8 days ago or sows (step 1) and those whose own piglets have just been weaned (step 2).
