# Supplementary material for: A mechanistic model for spread of livestock-associated methicillin-resistant Staphylococcus aureus (LA-MRSA) within a pig herd
Source: PLoS One. 2017 Nov 28;12(11):e0188429. doi: 10.1371/journal.pone.0188429 (PMC5705068; doi:10.1371/journal.pone.0188429)
Supplement: S2 Fig — (PDF) [file pone.0188429.s014.pdf]

S2 Fig. Model output: Convergence after introduction of one intermittently shedding gilt

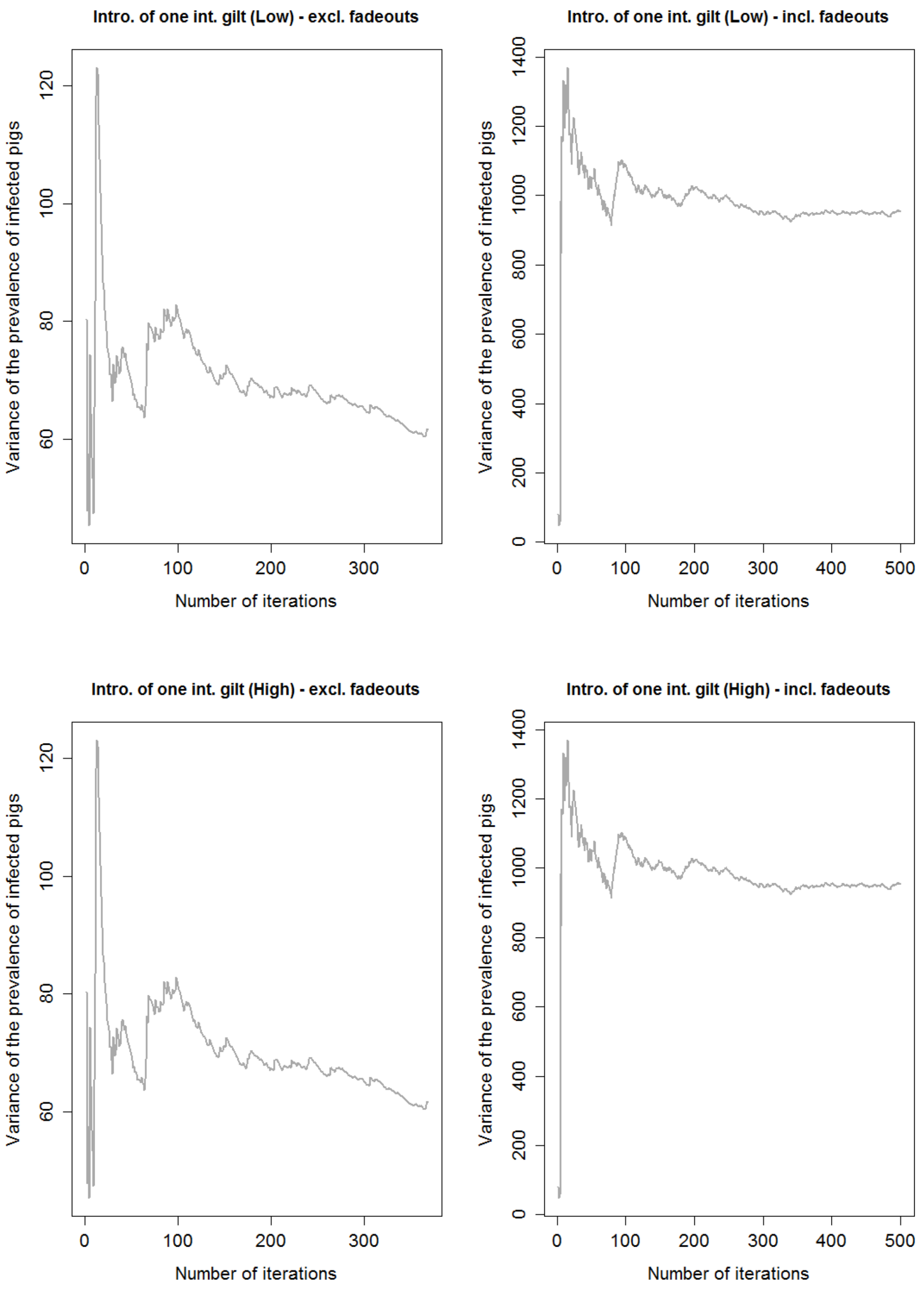

Note: All scenarios were run with 500 iterations. The number on the graphs to the left differ from 500, because iterations were MRSA faded out before the end of run were not included. It is assumed that convergence has been reached, when the variance stabilises.
