# Supplementary material for: A mechanistic model for spread of livestock-associated methicillin-resistant Staphylococcus aureus (LA-MRSA) within a pig herd
Source: PLoS One. 2017 Nov 28;12(11):e0188429. doi: 10.1371/journal.pone.0188429 (PMC5705068; doi:10.1371/journal.pone.0188429)
Supplement: S3 Fig — (PDF) [file pone.0188429.s015.pdf]

S3 Fig. Model output: Development in the median prevalence of MRSA shedders following introduction of one MRSA shedding weaner

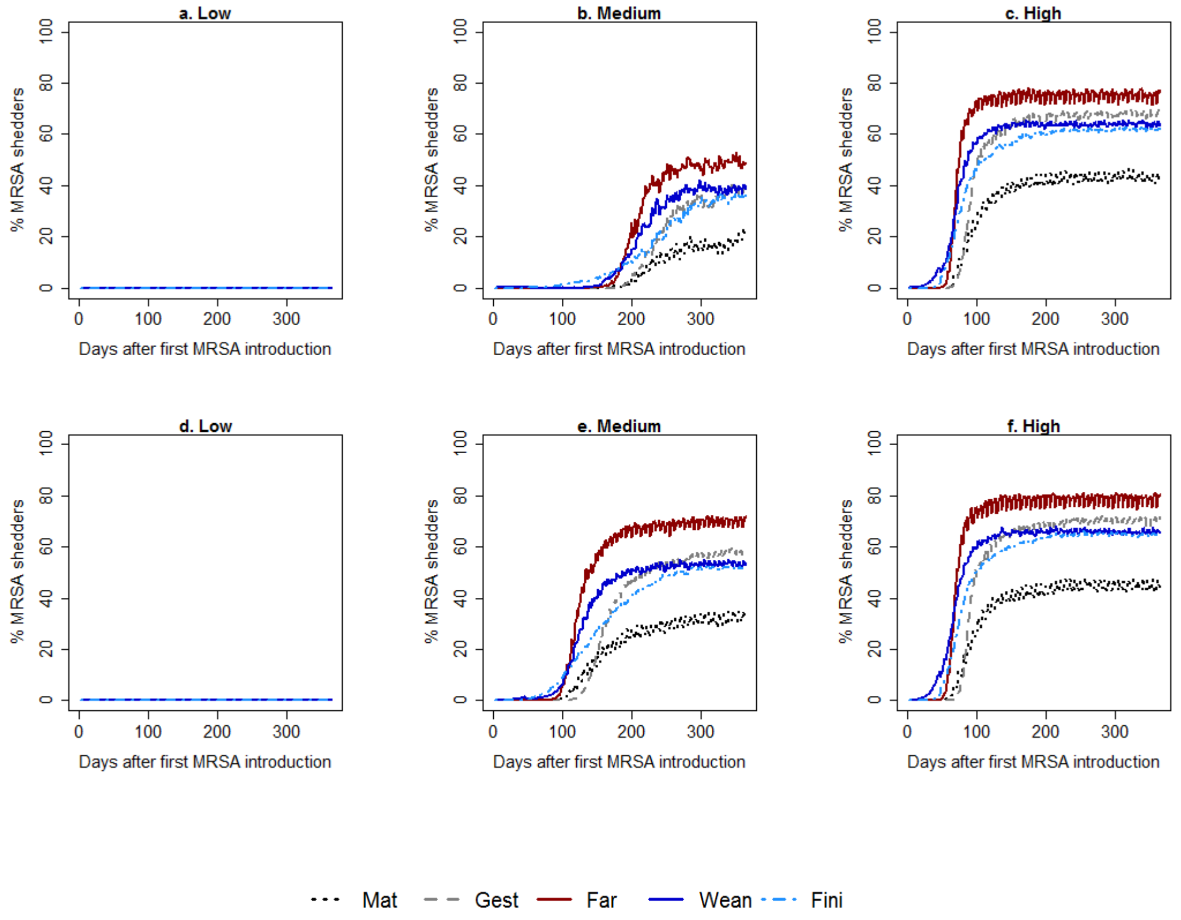

Predicted median prevalence over time following introduction of one intermittently (a-c) or persistently shedding weaner (d-f), when using low (a+d), medium (b+e) or high (c+f) transmission rates. Mat = Mating unit, Gest = Gestation unit, Farr = Farrowing unit, Wean = Weaner unit, Fini = Finisher unit. MRSA was introduced in the weaner section with the youngest pigs in order to ensure time for MRSA transmission to occur.
