# Supplementary material for: A mechanistic model for spread of livestock-associated methicillin-resistant Staphylococcus aureus (LA-MRSA) within a pig herd
Source: PLoS One. 2017 Nov 28;12(11):e0188429. doi: 10.1371/journal.pone.0188429 (PMC5705068; doi:10.1371/journal.pone.0188429)
Supplement: S6 Fig — (PDF) [file pone.0188429.s018.pdf]

S6 Fig. Model output: Violin plot of the prevalence following introduction of one finisher shedding MRSA intermittently or persistently

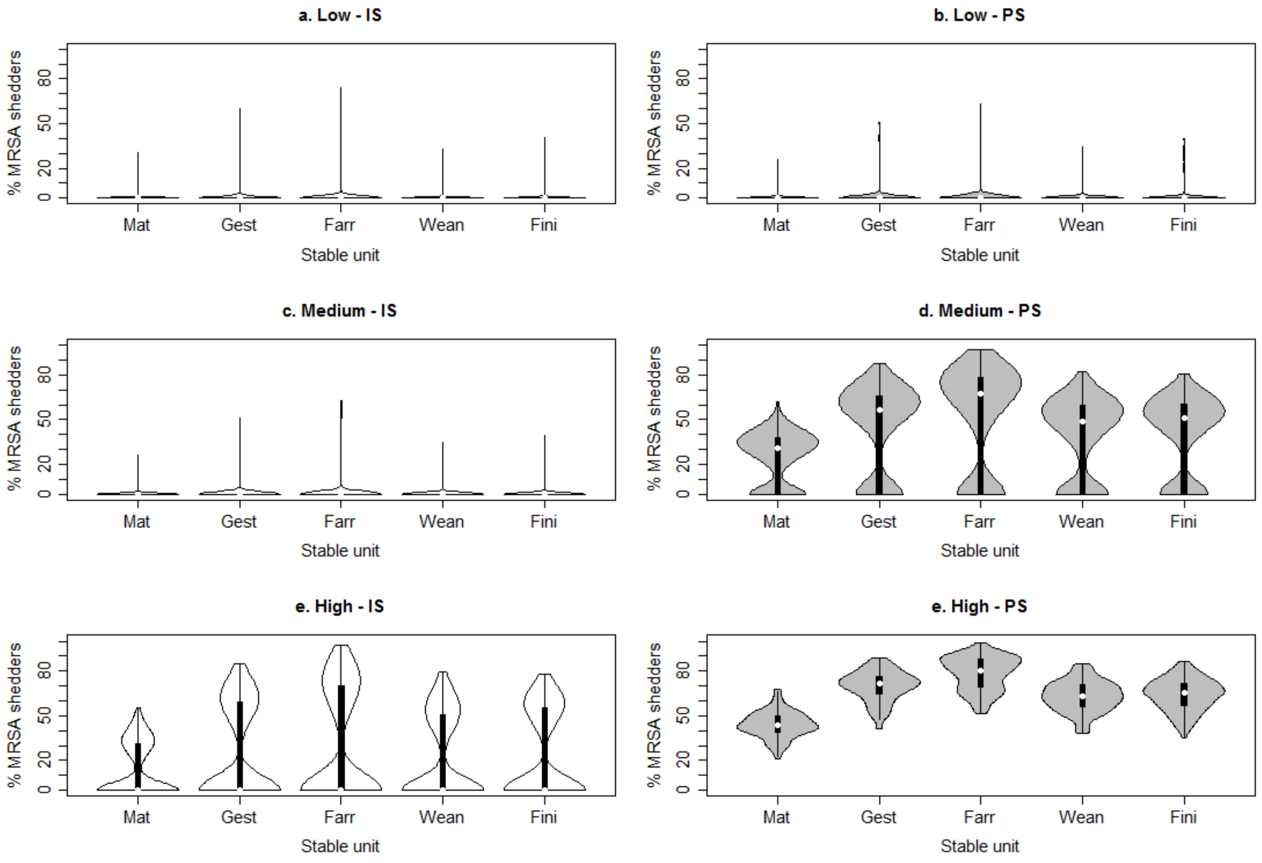

Predicted prevalence of MRSA shedders six years after introduction of a finisher shedding MRSA intermittently (left) or persistently (right) using low, medium or high transmission rates (distribution of 500 iterations). The median prevalences are indicated by white dots. Mat = Mating unit, Gest = Gestation unit, Farr = Farrowing unit, Wean = Weaner unit, Fini = Finisher unit.
