# Supplementary material for: A mechanistic model for spread of livestock-associated methicillin-resistant Staphylococcus aureus (LA-MRSA) within a pig herd
Source: PLoS One. 2017 Nov 28;12(11):e0188429. doi: 10.1371/journal.pone.0188429 (PMC5705068; doi:10.1371/journal.pone.0188429)
Supplement: S7 Fig — (PDF) [file pone.0188429.s019.pdf]

S7 Fig. Model output: Violin plot of the prevalence following introduction of one, ten or thirty finishers shedding MRSA intermittently

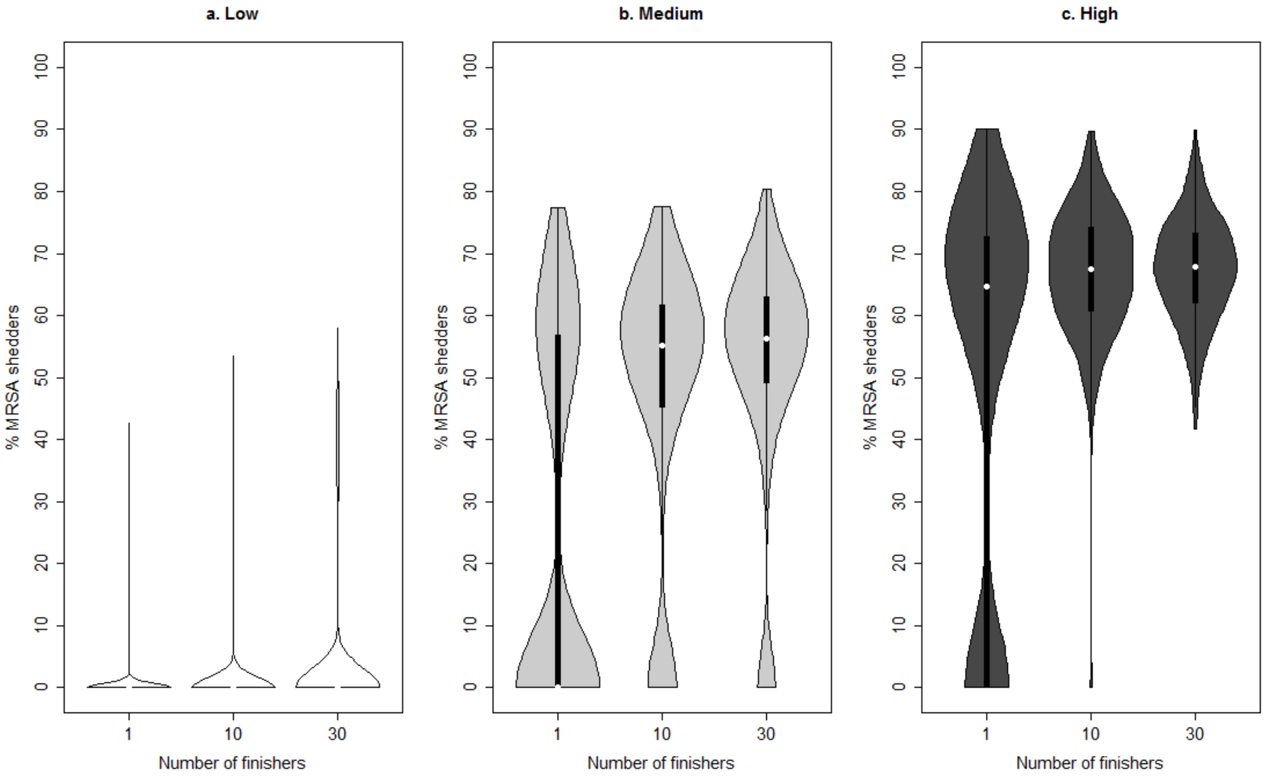

Predicted total prevalence of MRSA shedders in the herd six years after introduction of one, ten or thirty finishers shedding MRSA intermittently when low (a), medium (b) or high (c) transmission rates are used (distribution of 500 iterations). The median prevalences are indicated by white dots.
