# Supplementary material for: A mechanistic model for spread of livestock-associated methicillin-resistant Staphylococcus aureus (LA-MRSA) within a pig herd
Source: PLoS One. 2017 Nov 28;12(11):e0188429. doi: 10.1371/journal.pone.0188429 (PMC5705068; doi:10.1371/journal.pone.0188429)
Supplement: S8 Fig — (PDF) [file pone.0188429.s020.pdf]

S8 Fig. Model output: Development in the median prevalence of MRSA shedders following introduction of one, ten or thirty IS finishers

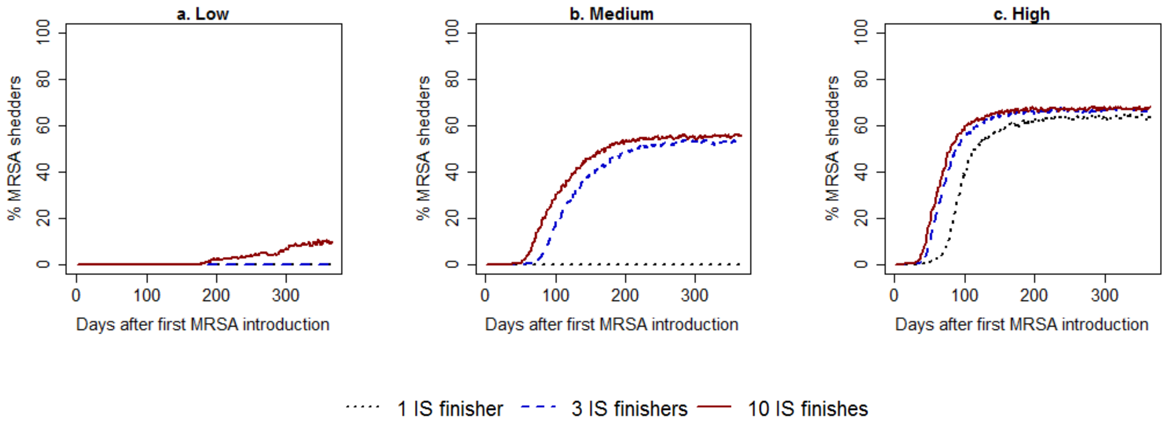

Predicted median prevalence over time when using low (a), medium (b) or high (c) transmission rates (distribution of 500 iterations).
