# Supplementary material for: A mechanistic model for spread of livestock-associated methicillin-resistant Staphylococcus aureus (LA-MRSA) within a pig herd
Source: PLoS One. 2017 Nov 28;12(11):e0188429. doi: 10.1371/journal.pone.0188429 (PMC5705068; doi:10.1371/journal.pone.0188429)
Supplement: S9 Fig — (PDF) [file pone.0188429.s021.pdf]

S9 Fig. Model output: Violin plot of the prevalence following introduction of one, three or ten gilt shedding MRSA intermittently every fortnight for three months

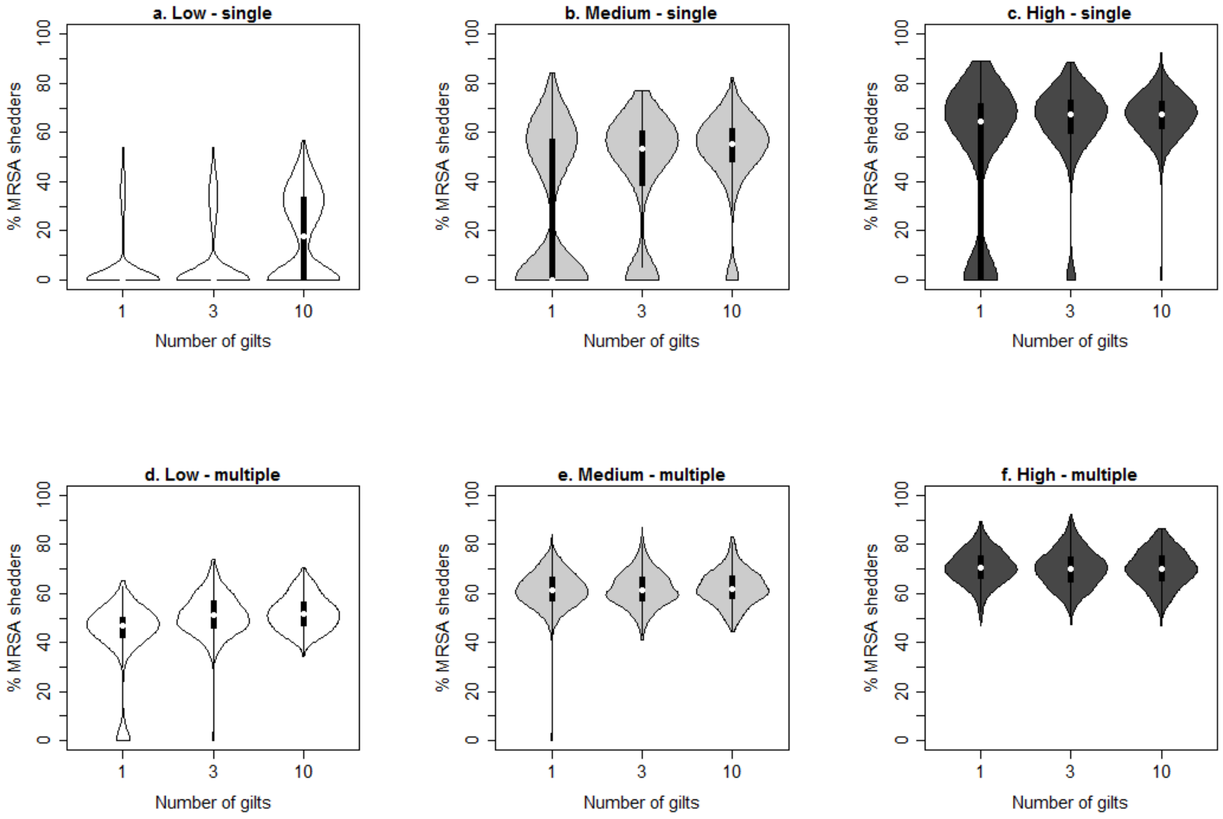

Predicted total prevalence of MRSA shedders in the herd six years after introduction of one, three or ten gilts shedding MRSA intermittently every fortnight for three months, when low (a), medium (b) or high (c) transmission rates are used (distribution of 500 iterations). The median prevalences are indicated by white dots.
