# Supplementary material for: A mechanistic model for spread of livestock-associated methicillin-resistant Staphylococcus aureus (LA-MRSA) within a pig herd
Source: PLoS One. 2017 Nov 28;12(11):e0188429. doi: 10.1371/journal.pone.0188429 (PMC5705068; doi:10.1371/journal.pone.0188429)
Supplement: S10 Fig — (PDF) [file pone.0188429.s022.pdf]

S10 Fig. Model output: Development in the median prevalence of MRSA shedders following single or multiple introductions

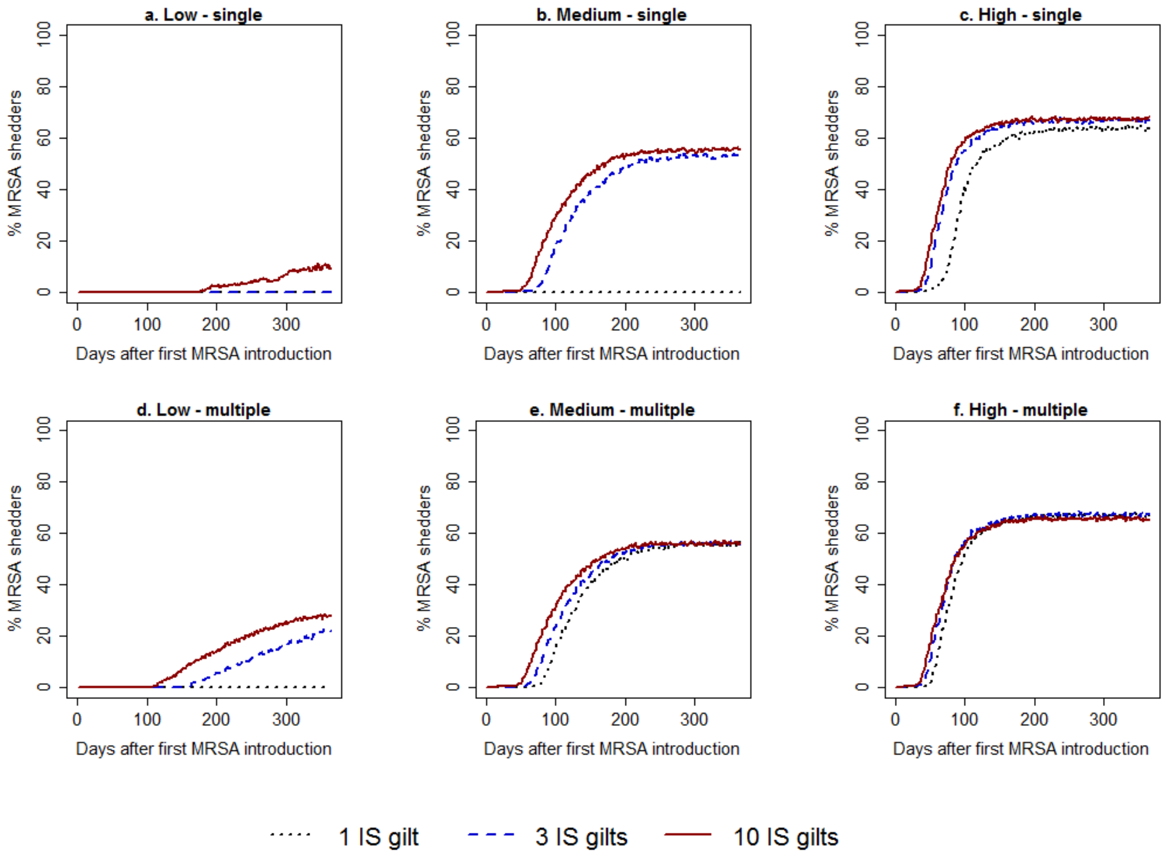

Predicted mean prevalence over time following single (a-c) or multiple (d-f) introductions of one, three or ten gilts shedding MRSA intermittently every fortnight for three months, when low (a+d), medium (b+e) or high (c+f) transmission was assumed.
